# Platelet methyltransferase-like protein 4-mediated mitochondrial DNA metabolic disorder exacerbates oral mucosal immunopathology in hypoxia

**DOI:** 10.1038/s41368-025-00373-9

**Published:** 2025-06-12

**Authors:** Yina Zhu, Meichen Wan, Yutong Fu, Junting Gu, Zhaoyang Ren, Yun Wang, Kehui Xu, Jing Li, Manjiang Xie, Kai Jiao, Franklin Tay, Lina Niu

**Affiliations:** 1 State Key Laboratory of Oral and Maxillofacial Reconstruction and Regeneration, National Clinical Research Center for Oral Diseases, Shaanxi Key Laboratory of Stomatology, Department of Prosthodontics, School of Stomatology, The Fourth Military Medical University, Xi’an, Shaanxi China; 2 Department of Aerospace Physiology, The Fourth Military Medical University, Xi’an, Shaanxi China; 3 Department of Stomatology, Tangdu Hospital, The Fourth Military Medical University, Xi’an, China; 4 The Dental College of Georgia, Augusta University, Augusta, GA USA

**Keywords:** Periodontitis, Innate immunity

## Abstract

Hypoxemia is a common pathological state characterized by low oxygen saturation in the blood. This condition compromises mucosal barrier integrity particularly in the gut and oral cavity. However, the mechanisms underlying this association remain unclear. This study used periodontitis as a model to investigate the role of platelet activation in oral mucosal immunopathology under hypoxic conditions. Hypoxia upregulated methyltransferase-like protein 4 (METTL4) expression in platelets, resulting in N^6^-methyladenine modification of mitochondrial DNA (mtDNA). This modification impaired mitochondrial transcriptional factor A-dependent cytosolic mtDNA degradation, leading to cytosolic mtDNA accumulation. Excess cytosolic mt-DNA aberrantly activated the cGAS-STING pathway in platelets. This resulted in excessive platelet activation and neutrophil extracellular trap formation that ultimately exacerbated periodontitis. Targeting platelet METTL4 and its downstream pathways offers a potential strategy for managing oral mucosa immunopathology. Further research is needed to examine its broader implications for mucosal inflammation under hypoxic conditions.

## Introduction

Hypoxemia, characterized by low oxygen blood levels,^1^ is commonly associated with high-altitude environments, respiratory diseases, and cardiovascular conditions.^2^ This condition affects 81.6 million individuals living at high altitudes and nearly 1 billion people with obstructive respiratory disorders.^3,4^ Research suggests a strong correlation between hypoxemia and mucosa barrier damage, particularly in the oral and gut mucosa.^5^ Epidemiological studies indicate that mucosal immunopathology in these regions worsens under hypoxic conditions.^5^ However, the underlying mechanism of this condition remains poorly understood.

Periodontitis, a prevalent oral mucosal disease worldwide, was chosen as the disease model in the present study to investigate the effects of hypoxia on mucosa immunopathology.^6^ In the present study, ribonucleic acid (RNA) sequencing identified a significant upregulation of genes associated with neutrophil activation and neutrophil extracellular trap (NET) formation under hypoxic conditions. NETs contribute to periodontitis in multiple ways through extracellular histones, reactive oxygen species (ROS) and proteolytic enzymes. More specifically, extracellular histone on NETs promotes IL17/Th17 axis and its downstream osteoclast differentiation, which derectly mediated alveolar bone loss in periodontitis.^7,8^ Although previous studies have linked NET formation in periodontitis to the oral microbiome, platelet activation, and fibrin accumulation,^7,9,10^ the mechanisms driving NET intensification under hypoxia remain unclear. Traditionally, platelets have been recognized for their roles in hemostasis and thrombosis.^11^ However, emerging evidence highlights their immune and inflammatory functions in various diseases. Activated platelets may induce NET formation and contribute to conditions such as sepsis and acute lung injury.^12,13^ Moreover, platelet activation has been implicated in hypoxia-related diseases.^14–16^ Based on these findings, we hypothesized that platelets respond to hypoxia by enhancing NET formation, ultimately causing mucosal barrier damage.

This study identified NETs as important contributors to the exacerbation of periodontitis under hypoxic conditions. Instead of directly responding to hypoxia, neutrophil-driven inflammation was upregulated by platelets. Hypoxia disrupted mitochondrial DNA (mtDNA) metabolism in platelets, resulting in platelet activation, neutrophil-platelet aggregation, and increased NET formation. This cascade ultimately aggravated periodontitis in hypoxic environments. These findings elucidate the mechanism by which hypoxia aggravates oral mucosal immunopathology and suggest novel therapeutic targets for its management. The findings also provide new insights into the potential role of platelet-mediated inflammation in other hypoxia-exacerbated mucosal diseases. Nevertheless, further research is needed to confirm this hypothesis.

## Results

### Effect of hypoxia on oral mucosa immunopathology in vivo

**Fig. 1:**
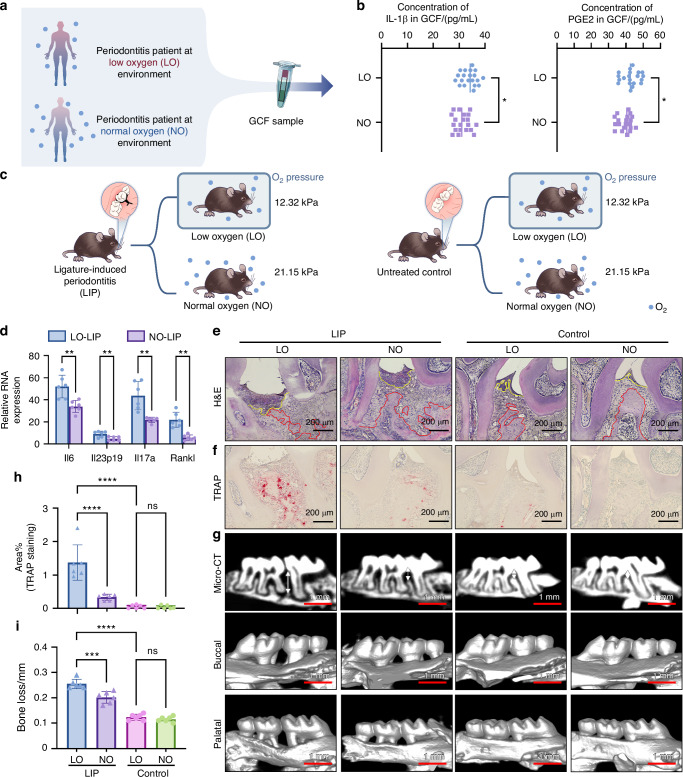
Hypoxia aggravates periodontitis in vivo. **a** Schematically depicting the procedure of human sample collection. **b** Concentration of IL-1β (left) and PGE2 (right) in the GCF of periodontitis patients in LO or NO environment (*n* = 20). **c** Schematically depicting the design of in vivo experiments. **d** Relative mRNA expression of inflammatory cytokines (*Il6*, *Il23p19*, *Il17* and *rankl*) in mouse gingiva subjected to LIP, under LO or NO environment (*n* = 6). **e** Representative images of H&E staining. Scale bars, 200 μm. The red dash represents the boundary between bone and connective tissue. The yellow dash represents the boundary between connective and epithelium. **f**, **g** Representative images of TRAP staining (**f**) and Micro-CT (**g**) of the periodontal tissue of mouse under LO or NO environment, with or without subjected to LIP. Scale bars of TRAP staining, 200 μm. Scale bars of Micro-CT, 1 mm. **h** Semi quantitative analysis of TRAP staining in **f** (*n* = 6). **i** Differences in bone loss among mouse in different groups (*n* = 6). The data are presented as the mean ± SD. ****P < 0.000 1; ***P < 0.001; **P < 0.01; *P < 0.05; ns, no significance. Statistical significance was determined by two-tailed unpaired Student’s *t* test (**b**, **d**) or one-way ANOVA test (**h**, **i**). IL-1β, Interleukin-1 β, PGE2 Prostaglandin E2, GCF gingival crevicular fluid, LO low oxygen, NO normal oxygen, H&E hematoxylin and eosin, TRAP tartrate resistant acid phosphatase, LIP ligature-induced periodontitis, micro-CT micro-computed tomography)

To investigate the relationship between hypoxia and oral mucosal inflammation, we first compared gingival crevicular fluid (GCF) from periodontitis patients in low-oxygen (LO; *n* = 20) and normal oxygen (NO; *n* = 20) environments. These patients were selected after carefully excluding known confounders or comorbidities. The definitions of LO and NO are detailed in the Methods section. The levels of interleukin-1β and prostaglandin E2 in GCF were measured. Results indicated significantly elevated concentrations of these inflammatory cytokines in LO patients (Fig. 1a, b).

To further examine this correlation, we established a murine ligature-induced periodontitis (LIP) model under hypobaric hypoxia (Fig. 1c). Consistent with the GCF findings, inflammatory gene expression was higher in LIP mice exposed to LO conditions (Fig. 1d). In addition, LO-exposed LIP mice exhibited increased bone destruction and more severe periodontitis, whereas LIP mice in NO conditions experienced minimal disease progression (Fig. 1e–i). These findings suggest that hypoxic environments exacerbate periodontitis. Further research is required to elucidate the precise mechanism by which hypoxia contributes to disease progression.

Since the oral microbiome plays an important role in periodontitis, we next examine whether hypoxia-induced changes in the microbiome contribute to disease exacerbation. 16S rRNA gene sequencing was used to characterize the microbiome. However, microbiome composition and structure showed no significant differences between LO-LIP and NO-LIP mice (Fig. S1a–c). Although previous studies have reported substantial microbiome shifts under hypoxic conditions such as high altitude or obstructive sleep apne,^17,18^ it is important to note that oxygen levels in periodontal pockets remain inherently low even in common forms of periodontitis.^19^ This may explain the minimal microbiome alterations observed in this study.

To further dissect microbiome contributions, we eliminated dental plaque using antibiotic-treated ligatures (Fig. S1d). Despite plaque elimination, periodontitis symptoms remained aggravated under hypoxia compared to antibiotic-treated mice in NO conditions (Fig. S1e–g). These findings suggest that the oral microbiome is not the primary mediator of hypoxia-aggravated mucosal immunopathology. Instead, hypoxia may exacerbate periodontitis through alternative mechanisms.

### Role of NETosis in exacerbating oral mucosal lesions under hypoxia

**Fig. 2:**
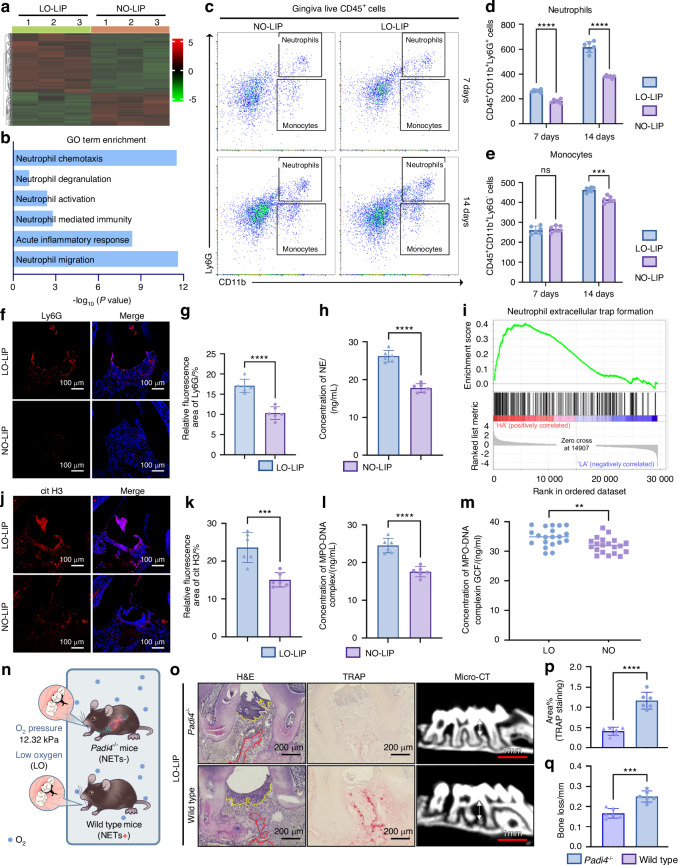
The effect of NETs on aggravates periodontitis under hypoxic condition. **a** Heatmap of the differential genes. **b** Enriched gene ontology terms comparing LO-LIP and NO-LIP groups. **c** Flow cytometry analysis of gingiva tissues from LO-LIP and NO-LIP mouse. **d**, **e** Absolutely count of neutrophils (**d**) and monocytes (**e**) in mouse gingiva (*n* = 6). **f** Representative CLSM images of periodontal tissues from LO-LIP and NO-LIP mice which were stained with Ly6G and DAPI. Scale bars, 100 μm. **g** Semi-quantitative analysis of Ly6G signaling in **e** (*n* = 6). **h** Concentration of NE in the lysate of mouse periodontal tissue (*n* = 6). **i** GSEA plot of the upregulated NETs formations KEGG pathways in LO-LIP groups. **j** Representative CLSM images of periodontal tissues from LO-LIP and NO-LIP mice, which was stained with cit H3 and DAPI. Scale bars, 100 μm. **k** Semi-quantitative analysis of cit H3 signaling in (**i**) (*n* = 6). Concentration of MPO-DNA complex in the lysate of mouse periodontal tissue (*n* = 6) (**l**) and the GCF of periodontitis patients (*n* = 20) (**m**). **n** Schematically illustrating the design of in vivo experiments. **o** Representative images of H&E staining (left), TRAP staining (middle) and micro-CT (left) of the periodontal tissue of *Padi4*^*-/-*^ mice and wild type mice. The red dash represents the boundary between bone and connective tissue. The yellow dash represents the boundary between connective tissue and epithelium. Scale bars of H&E staining and TRAP staining, 200 μm. Scale bars of micro-CT, 1 mm. **p** Semi quantitative analysis of TRAP staining in (**o**) (*n* = 6). **q** Bone loss measurement of *Padi4*^*−/−*^ mice and wild type mice (*n* = 6). The data are presented as the mean ± SD. ****P < 0.000 1; ***P < 0.001; **P < 0.01; *P < 0.05; ns, no significance. Statistical significance was determined by two-tailed unpaired Student’s *t* test. NETs neutrophil extracellular traps, MPO-DNA myeloperoxidase-deoxyribonucleic acid, CLSM confocal laser scanning microscopy, GCF gingival crevicular fluid, NE neutrophil elastase, cit H3 citrullinated histone H3, H&E hematoxylin and eosin, LIP ligature-induced periodontitis, TRAP Tartrate resistant acid phosphatase, micro-CT micro-computed tomography, LO low oxygen, NO normal oxygen)

Progression of oral mucosal immunopathology is not solely related to the oral microbiome; it is also influenced by a dysfunctional immune response. Having excluded microbiome changes, we explored the the role of the immune system in hypoxia-aggravated mucosal immunopathology. Mice were subjected to LIP and housed in either low-oxygen (LO-LIP) or normal-oxygen (NO-LIP) environments. Gingival tissues derived from these mice that underwent RNA sequencing revealed significant gene expression differences between the LO-LIP and NO-LIP groups (Fig. 2a). Gene ontology analysis showed a marked upregulation of pathways related to neutrophil activation and neutrophil-mediated inflammatory responses in LO-LIP mice (Fig. 2b).

Consistent with transcriptomic data, flow cytometry demonstrated increased neutrophil infiltration (live CD45^+^CD11b^+^Ly6G^+^) in both early and late stages of gingival inflammation in LO-LIP mice (Fig. 2c, d). In contrast, other inflammatory cells including monocytes (live CD45^+^CD11b^+^Ly6G^-^), B cells (live CD45^+^B220^+^), and T cells (live CD45^+^TCRβ^+^) only demonstrated increased infiltration at later stages (Fig. 2e, Fig. S2). Immunostaining for Ly6G and elevated neutrophil elastase further validated these findings (Fig. 2f–h). Collectively, these findings suggest that neutrophils play a central role in exacerbating periodontitis under hypoxic conditions. However, the precise mechanism by which neutrophils contribute to disease progression requires further investigation.

Further analysis of RNA-seq data was conducted to examine the role of neutrophils in hypoxia-aggravated periodontitis. Gene Set Enrichment Analysis revealed an upregulation of NET formation in LO-LIP mice (Fig. 2i). LO-LIP mice showed elevated NET markers-citrullinated histone H3 (citH3) and myeloperoxidase (MPO)-DNA complexes in gingival tissues (Fig. 2j–l). This finding was also confirmed in human GCF samples (Fig. 2m). These results suggest that NETosis plays an indispensible role in the exacerbation of periodontitis under hypoxic conditions. To functionally validate NETosis’ role, we used *Padi4*^−/−^ mice, *peptidyl arginine deiminase type 4* (*Padi4*)-deficient mice (*Padi4*^−*/*−^) that lack the ability to form NETs were used to validate this hypothesis. Following a 14-day exposure to LIP in LO environment (Fig. 2n), *Padi4*^−*/*−^ mice exhibited significantly reduced periodontitis severity (Fig. 2o, p). Degradation of existing NETs using DNase I also alleviated periodontitis under hypoxia (Fig. S3). These findings indicate that excessive NET formation under hypoxic conditions contributes significantly to oral mucosal inflammation. However, the precise mechanisms driving increased NETosis in hypoxia remain to be elucidated.

### Effect of hypoxia on platelet-induced NETosis

Bacteria are potent stimuli for NET formation in common forms of periodontitis.^10^ Periodontal pathobionts such as *Porphyromonas gingivalis* (Pg), have been shown to directly induce NET formation.^20,21^ However, oral microbiome specimens from LO-LIP and NO-LIP mice showed no significant differences in their ability to elicit NET formation (Fig. S4). This prompted further investigation into whether hypoxia directly induces NET release and contributes to periodontitis progression.

To examine this notion, neutrophils from mice adapted to an LO environment for 7 days (LO-Neu) and normoxic controls (NO-Neu) were collected and cultured in a medium containing inactivated Pg. The aforementioned experiment was conducted to simulate the periodontitis microenvironment in vitro. Both LO-Neu and NO-Neu were incubated under hypoxic and normoxic conditions, respectively. Evaluation of NET formation indicated no significant differences between the two groups (Fig. S5a, b), suggesting that hypoxia alone does not directly enhance NET formation.

The potential role of hypoxia-exposed neutrophils (LO-Neu) directly exacerbates periodontitis was further examined in an in vivo model. This model comprised using mice that express the diphtheria toxin receptor (DTR) under the control of the Ly6G promotor that is expressed specifically in neutrophils (Ly6G-DTR mice) to achieve neutrophil depletion.^22^ Host NO-Neu were depleted before subjecting the mice to LIP under normoxic conditions. Daily DT injections were used to deplete endogenous neutrophils, and LO-Neu or NO-Neu from donor mice was transfused to evaluate their effects on periodontitis (Fig. S5c). The results showed that LO-Neu failed to aggravate periodontitis compared to NO-Neu (Fig. S5d–f). This observation further indicates that hypoxia does not directly exacerbates neutrophil-mediated periodontitis.

**Fig. 3:**
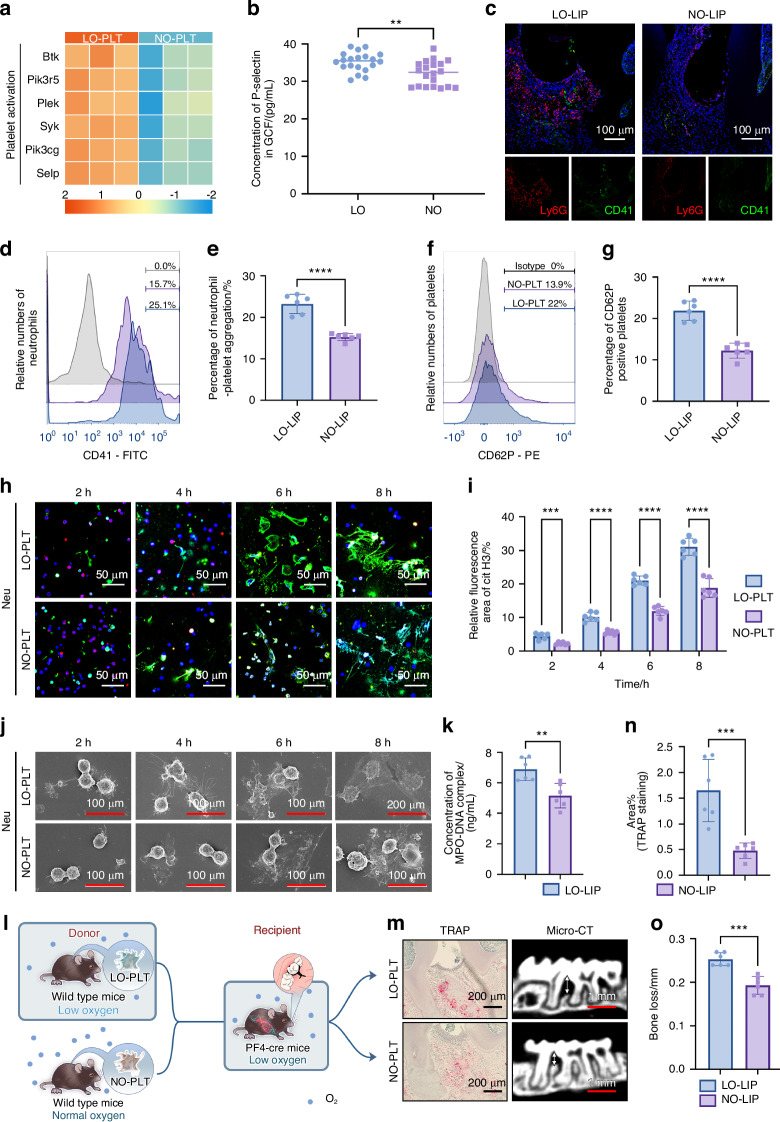
Platelets mediated NETosis upregulation under hypoxic condition. **a** Heatmap of representative differentially expressed genes related to platelet activation. **b** Concentration of P-selectin in the GCF of periodontitis patients (*n* = 20). **c** CLSM images of periodontal tissues from LO-LIP and NO-LIP mice, which were stained with Ly6G (red) and CD41 (green). Scale bars, 100 μm. **d** Flow cytometry analysis of neutrophil-platelet aggregation. **e** Changes of neutrophil-platelets aggregation in percentage (*n* = 6). **f** Flow cytometry analysis of P-selectin positive platelets. **g** Changes of P-selectin positive platelets in percentage (*n* = 6). **h**, **j** Representative CLSM (**h**) and SEM (**j**) images of neutrophil incubated with LO-PLT and NO-PLT for 2 h, 4 h, 6 h, and 8 h. Scale bars of CLSM, 50 μm. **i** Semi-quantitative of cit H3 signaling in (**h**) (*n* = 6). **k** Concentration of MPO-DNA complex in the supernatant of neutrophils incubated with LO-PLT or NO-PLT (*n* = 6). **l** Schematic illustrating the design of in vivo experiments. **m** Representative TRAP staining and micro-CT images of the periodontal tissue of PF4-DTR mice transfused with LO-PLT and NO-PLT. Scale bars of TRAP staining, 200 μm. Scale bars of micro-CT 1 mm. **n** Semi-quantitative analysis of TRAP staining in (**m**) (*n* = 6). **o** Bone loss measurement of differently treated PF4-DTR mice (*n* = 6). The data are presented as the mean ± SD. ****P < 0.000 1; ***P < 0.001; **P < 0.01. Statistical significance was determined by two-tailed unpaired Student’s *t* test. MPO-DNA myeloperoxidase-deoxyribonucleic acid, CLSM confocal laser scanning microscopy, GCF gingival crevicular fluid, NE neutrophil elastase, PLT platelets, cit H3 citrullinated histone H3, TRAP Tartrate resistant acid phosphatase, LIP ligature-induced periodontitis, micro-CT micro-computed tomography, LO low oxygen, NO normal oxygen, Neu neutrophils

Platelet activation plays a critical role in the pathogenesis of hypoxia-related diseases and contributes to NET formation.^12,13,15^ Hence, it is logical to examine whether platelets respond to hypoxia by upregulating NET formation and exacerbating oral mucosal inflammation. Microarray analysis of blood samples from healthy adults transported to high altitudes revealed upregulation of pathways related to platelet aggregation.^23^ Further RNA sequencing analysis in the present study confirmed increased expression of genes associated with platelet activation (Fig. 3a). Consistent results observed in GCF samples collected from periodontitis patients reinforced the presence of platelet activation-related gene signatures (Fig. 3b). These findings suggest that platelet activation may contribute to periodontitis exacerbation under hypoxic conditions. Increased platelet and neutrophil infiltration in the gingival tissue of LO-PLT mice further indicated a potential interaction between these cell types (Fig. 3c). Flow cytometry analysis demonstrated a high percentage of neutrophil-platelet aggregation in the blood of LO-LIP mice (Fig. 3d, e) and higher percentage of activated platelets from LO-LIP mice (Fig. 3f, g). These findings suggest that hypoxia may influence platelet activation and promote neutrophil-platelet aggregation.

To examine whether increased neutrophil-platelet aggregation enhances NET formation, platelets from LO-LIP and NO-LIP mice were incubated with neutrophils in vitro. Over time, LO-PLT significantly promoted NET formation (Fig. 3h–k), suggesting that hypoxia may drive NETosis through platelet activation. To directly test platelet involvement, platelet Factor 4 (PF4)-DTR mice were used to further confirm the role of LO-PLT in exacerbating periodontitis under hypoxic conditions.^24^ Platelets were depleted using DT, after which the mice underwent LIP in a hypoxic environment and received transfusions of either LO-PLT or NO-PLT (Fig. 3l). The PF4-DTR mice transfused with NO-PLT showed alleviated periodontitis symptoms (Fig. 3m–o). Taken together, these observations indicate that hypoxia upregulated platelet activation, enhances neutrophil-platelet aggregation, and drives NETosis to exacerbatr periodontitis. Further research is needed to elucidate the precise mechanisms by which hypoxia induces platelet activation and NET formation.

### Contribution of hypoxia to platelet cGAS-STING signaling and NET formation

In the procedure of platelets activation mediated NETs formation, the cyclic guanosine monophosphate-adenosine monophosphate synthase (cGAS)-stimulator of interferon genes (STING) pathway plays a profound role.^25^ To be specifically, the product of cGAS activation-cyclic guanosine monophosphate-adenosine monophosphate (cGAMP) induces palmitoylation of STING, enabling its interaction with syntaxin binding protein 2 (STXBP2) to mediate platelet activation and NET formation.4 Although previous study have linked cGAS-STING pathway activation to hypoxia-induced disease, whether hypoxia upregulate cGAS-STING pathway and by which mechanism remain obscure.^9^ Therefore, we hypothesize that hypoxia exacerbates cGAS-STING activation in platelets, thereby enhancing downstream effects such as NET formation.

**Fig. 4:**
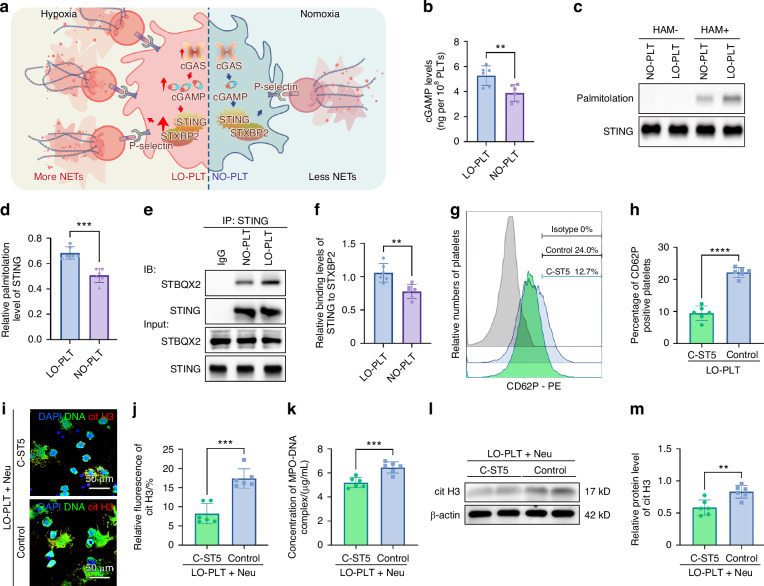
Hypoxia amplifies platelet cGAS-STING signaling to enhance NETs formation. **a** Schematic illustration of the overall mechanism in this figure. **b** Concentration of cGAMP in the cytoplasm from 10^8^ platelets (*n* = 6). **c** The level of STING palmitoylation in LO-PLT and NO-PLT after incubated with *Pg* medium. **d** Quantitative analysis of the gray values of STING palmitoylation in (**c**) (*n* = 6). **e** IP-STING assay of LO-PLT and NO-PLT after incubated with *Pg* medium. Isotype-matched IgG served as the negative control. **f** Quantitative analysis of STBQX2 bands in (**e**) (*n* = 6). **g** Flow cytometry analysis of P-selectin positive platelets. **h** Changes of P-selectin positive platelets in percentage (*n* = 6). **i** Representative CLSM images of neutrophils incubated with C-ST5 treated LO-PLT or untreated LO-PLT. Scale bars, 50 μm. **j** Semi-quantitative of cit H3 signaling in (**i**) (*n* = 6). **k** Concentration of MPO-DNA complex in the supernatant of neutrophils incubated with C-176 treated HO-PLT or untreated HO-PLT (*n* = 6). **l** Expression of cit H3 in neutrophils incubated with C-ST5 treated LO-PLT or untreated LO-PLT. **m** Semi-quantitative analysis of cit H3 in (**l**) (*n* = 6). The data are presented as the mean ± SD. ****P < 0.000 1; ***P < 0.001; **P < 0.01; ns, no significance. Statistical significance was determined by two-tailed unpaired Student’s *t* test. MPO-DNA myeloperoxidase-deoxyribonucleic acid, CLSM confocal laser scanning microscopy, cit H3 citrullinated histone H3, PLT platelets, LO low oxygen, NO normal oxygen, Neu neutrophils, cGAMP cyclic guanosine monophosphate-adenosine monophosphate, HAM hydroxylamine, STING stimulator of interferon genes, STXBP2 syntaxin binding protein 2

Evaluation of cGAS-STING pathway activation in LO-PLT and NO-PLT revealed a significant increase in cytosolic levels of cGAMP, the product of cGAS, in platelets exposed to hypoxia (Fig. 4b). Consistent with this observation, LO-PLT exhibited elevated STING palmitoylation and enhanced binding to syntaxin binding protein 2 (Fig. 4c–f). These findings suggest that hypoxia enhances activation of the platelet cGAS-STING pathway.

To further elucidate the functional role of the cGAS-STING pathway in platelet activation under hypoxic conditions, the C-ST5 peptide, a platelet-specific inhibitor of this pathway, was employed. Inhibition of STING binding to syntaxin binding protein 2 with C-ST5 significantly reduced LO-PLT activation (Fig. 4g, h) and concurrently weakened NET formation (Fig. 4i–m). These results highlight the role of the cGAS-STING pathway in platelet activation and NET formation under hypoxia. This prompted further investigation into the mechanisms driving cGAS-STING upregulation in platelets under hypoxic conditions.

### Effect of hypoxia on mtDNA metabolism in platelets

**Fig. 5:**
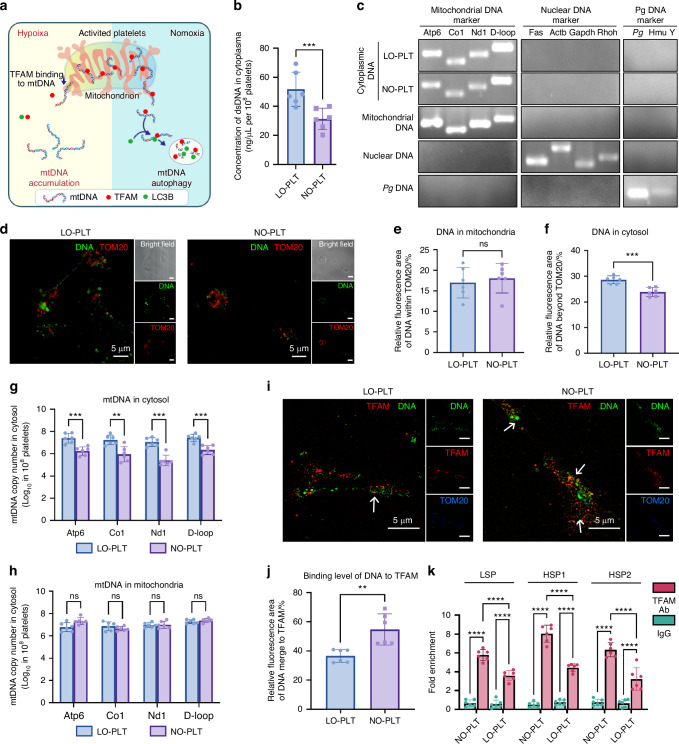
TFAM-dependent cytosolic mtDNA degradation is inhibited in platelets under hypoxic condition. **a** Schematic illustration of the overall mechanism in this figure. **b** Concentration of dsDNA in the cytoplasm from 10^8^ platelets (*n* = 6). **c** Images from agarose gel electrophoresis of the DNA extracted from cytoplasm of LO-PLT and NO-PLT incubated with *Pg* medium. Mitochondrial DNA isolated from platelets, nuclear DNA isolated from neutrophils and *Pg* DNA isolated from *Pg* were used as controls. **d** CLSM images of LO-PLT and NO-PLT incubated with *Pg* medium, stained with DNA (green) and TOM20 (red). Scale bars, 5 μm. **e**, **f** Semi-quantitative of the DNA signaling not merged with TOM20 (**e**) and the DNA signaling merged with TOM20 (**f**) in (**d**) (*n* = 6). **g**, **h** Quantification of mtDNA copy number by PCR using the probe of *Atp6*, *Co1*, *Nd1* and *D-loop*, from isolated cytosolic fractions (**g**) or mitochondrial fractions (**h**) of LO-PLT and NO-PLT incubated with *Pg* medium (*n* = 6). **i** CLSM images of LO-PLT and NO-PLT incubated with *Pg* medium, stained with DNA (green), TFAM (red) and TOM20 (blue). Scale bars, 5 μm. **j** Semi-quantitative of the DNA signaling that merged with TFAM in (**l**) (*n* = 6). **k** Chromatin immunoprecipitation assay was conducted to examine the occupancy of TFAM within mtDNA promoter regions, including HSP1, HSP2, and LSP, in LO-PLT and NO-PLT incubated with *Pg* medium (*n* = 6). The data are presented as the mean ± SD. ****P < 0.000 1; ***P < 0.001; **P < 0.01; ns, no significance. Statistical significance was determined by two-tailed unpaired Student’s *t* test (**b**, **e**, **f**, **g**, **h**, **j**) or two-way ANOVA test (**k**). CLSM confocal laser scanning microscopy, LO low oxygen, NO normal oxygen, PLT platelets, TFAM mitochondrial transcriptional factor A, LC3B microtubule-associated protein 1 light chain 3, mtDNA, *Pg Porphyromonas gingivalis*, TOM20 translocase of outer mitochondrial membrane 20, mtDNA mitochondrial DNA

cGAS functions as a cytosolic DNA sensor and is activated by elevated levels of double-stranded DNA (dsDNA) in the cytoplasm.^26^ Consistent with this mechanism, an increased concentration of cytosolic DNA was consistently observed in LO-PLT (Fig. 5b), which may contribute to abnormal cGAS activation. Platelets contain both endogenous and exogenous dsDNA, with endogenous dsDNA originating from mitochondria (mtDNA) and exogenous dsDNA derived from ingested fragments of nucleated cells or bacteria.^27^ Therefore, identifying the source of the increased cytosolic DNA was necessary.

To elucidate the source of the increased cytosolic DNA, DNA was extracted from platelets, amplified, and analyzed using polymerase chain reaction (PCR). Agarose gel electrophoresis revealed that cytosolic DNA in LO-PLT and NO-PLT primarily originated from mitochondria rather than external sources (Fig. 5c). These findings explain the upregulation of the cGAS-STING pathway and the accumulation of excess cytosolic DNA. However, the mechanism by which hypoxia mediated the increase of cytosolic mtDNA in platelets requires further investigation.

Given that platelets are anucleate, the increased concentration of mtDNA in their cytoplasm may arise from either enhanced mtDNA leakage from mitochondria or impaired mtDNA degradation. Immunofluorescence analysis showed no significant changes in mitochondrial DNA content, as indicated by the colocalization of red and green fluorescent signals (Fig. 5d, e). However, an increase in cytosolic DNA was observed in LO-PLT, represented by non-co-localized green signals (Fig. 5d, f). These observations were further confirmed by quantitative PCR (Fig. 5g, h).

Moreover, mitochondrial reactive oxygen species (ROS), which are implicated in mtDNA leakage in activated platelets, showed no significant differences between LO-PLT and NO-PLT.^28^ Furthermore, the integrity of mtDNA in LO-PLT was also comparable to that of the control group (Fig. S6). These results indicate that the accumulation of cytosolic mtDNA is not attributable to mitochondrial leakage but rather to impaired mtDNA degradation under hypoxic conditions.

Cytosolic mtDNA degradation is regulated by two distinct pathways. The first involves non-specific degradation mediated by three prime repair exonuclease 1 (TREX1), while the second relies on an autophagy pathway driven by mitochondrial transcriptional factor A (TFAM).^29,30^ However, the expression levels of both TREX1 and TFAM remained unchanged between LO-PLT and NO-PLT (Figure. S7).

Since TFAM-dependent mtDNA degradation requires its binding to mtDNA,^30^ immunofluorescence staining was performed to examine TFAM and DNA co-localization in platelets. The results showed a significant reduction in TFAM-mtDNA co-localization in LO-PLT (Fig. 5i, j), suggesting impaired mtDNA degradation in the cytoplasm. This observation was further supported by chromatin immunoprecipitation, which confirmed a weaker interaction between TFAM and mtDNA in LO-PLT (Fig. 5k).

These findings reveal a mechanism underlying impaired cytosolic mtDNA degradation in platelets under hypoxic conditions: TFAM dissociation from mtDNA disrupts the TFAM-mediated degradation pathway. Consequently, the accumulation of cytosolic mtDNA upregulates the cGAS-STING signaling pathway to enhance platelet activation and NET formation (Fig. 5a). Further investigation is needed to determine how hypoxia disrupts TFAM-mtDNA binding.

Two factors inhibit the interaction between mtDNA and TFAM. The first factor is mtDNA modification, specifically N^6^-methyladenine (6 mA). This is the only known modification that interferes with TFAM binding to mtDNA to interfere with its degradation.^31^ The second factor involves TFAM modifications such as phosphorylation and acetylation.^32^ However, studies on TFAM modifications have yielded conflicting results. Whereas one study reported that phosphorylated or acetylated TFAM has reduced affinity for mtDNA in vitro,^32^ a more recent study found that these modifications do not affect TFAM-mtDNA binding.^33^

**Fig. 6:**
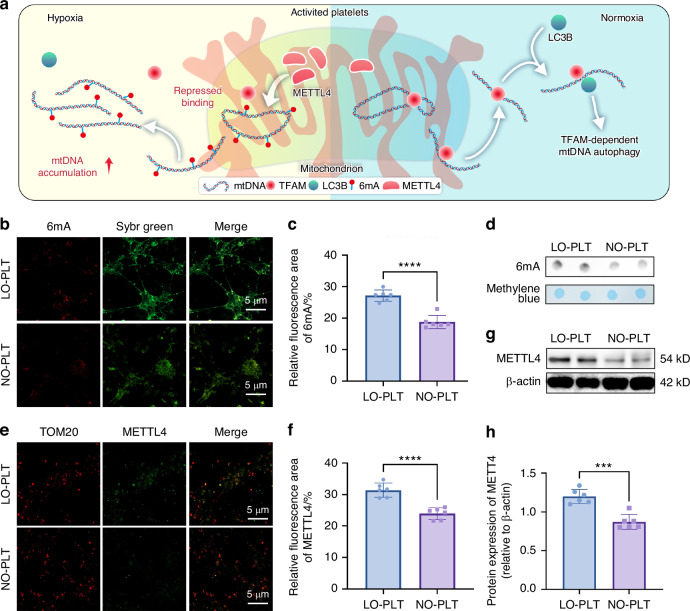
Hypoxia promotes mtDNA 6 mA methylation via METTL4 in platelets. **a** Schematic illustration of the overall mechanism in this figure. **b** CLSM images of LO-PLT and NO-PLT stained with 6 mA (red) and sybr green (green). Scale bars, 5 μm. **c** Semi-quantitative of the 6 mA signaling in (**b**) (*n* = 6). **d** mtDNA 6 mA levels of LO-PLT and NO-PLT were assessed by dot blot analysis. **e** CLSM images of LO-PLT and NO-PLT stained with METTL4 (green) and TOM20 (red). Scale bars, 5 μm. **f** Semi-quantitative of the 6 mA signaling in (**e**) (*n* = 6). **g** The expression of METTL4 in LO-PLT and NO-PLT. **h** Semi-quantitative of METTL4 in (**g**) (*n* = 6). The data are presented as the mean ± SD. ****P < 0.000 1; ***P < 0.001. Statistical significance was determined by two-tailed unpaired Student’s *t* test. CLSM confocal laser scanning microscopy, LO low oxygen, NO normal oxygen, PLT platelets, mtDNA mitochondrial DNA, 6 mA N^6^-methyladenine, LC3B microtubule-associated protein 1 light chain 3, METTL4 methyltransferase-like protein 4

To address these discrepancies, the impact of hypoxia on mtDNA 6 mA levels in platelets was investigated. The significant increase in mtDNA 6 mA in LO-PLT (Fig. 6b–d) suggests that elevated 6 mA methylation may inhibit mtDNA binding to TFAM under hypoxia. Methyltransferase-like protein 4 (METTL4), a eukaryotic homolog of the bacterial 6 mA methyltransferase DNA adenine methyltransferase 1, is known to catalyze 6 mA methylation in mammalian DNA.^31^ A previous study reported that METTL4 expression is significantly upregulated through p53 activation when cardiomyocytes are exposed to hypoxia.^34^ A similar mechanism may occur in platelets. Indeed, METTL4 was specifically localized to platelet mitochondria (Fig. 6e), and its expression was significantly upregulated in LO-PLT (Fig. 6e–h). These findings indicate that hypoxia upregulated METTL4 in platelets, facilitates mtDNA 6 mA methylation, disrupts TFAM-dependent mtDNA degradation, and results in cytoplasmic mtDNA accumulation (Fig. 6a).

### Impact of platelet METTL4 depletion on periodontitis in hypoxia

**Fig. 7:**
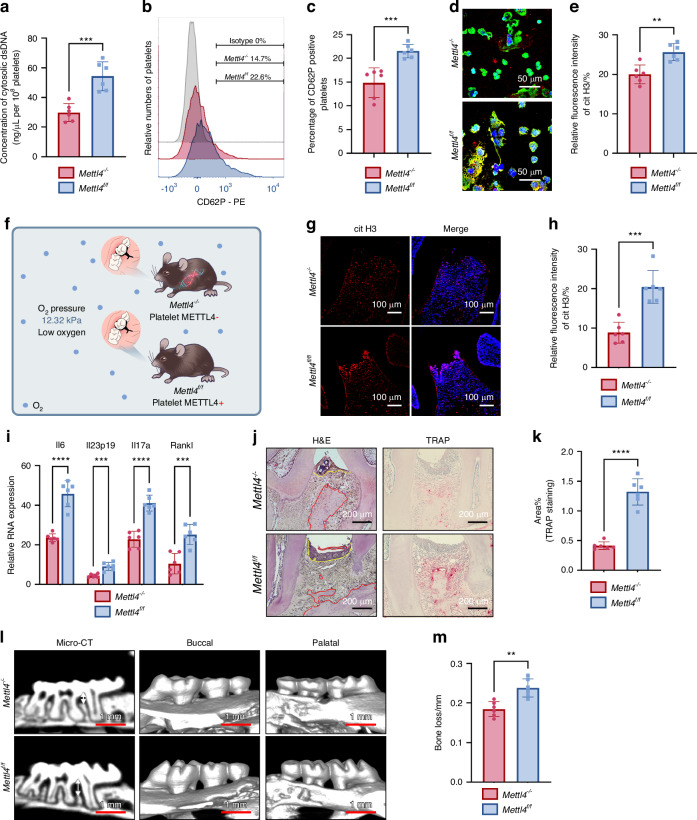
Platelet METTL4 deficiency alleviates periodontitis under hypoxic condition. **a** Concentration of cytosolic dsDNA in LO-PLT derived from *Mettl4*^*−/−*^ and *Mettl4*^*f/f*^ (*n* = 6). **b** Flow cytometry analysis of P-selectin positive platelets. **c** Changes of P-selectin positive platelets in percentage (*n* = 6). **d** CLSM images of neutrophils incubated with LO-PLT derived from *Mettl4*^*−/−*^ and *Mettl4*^*f/f*^. Scale bars, 50μm. **e** Semi-quantitative of the cit H3 signaling in (**d**) (*n* = 6). **f** Schematic design of the in vivo experiment. **g** Representative CLSM images of periodontal tissues of *Mettl4*^*−/−*^ and *Mettl4*^*f/f*^ mice that subjected to LO-LIP, stained with cit H3 and DAPI. Scale bars, 100 μm. **h** Semi-quantitative of the cit H3 signaling in (**g**) (*n* = 6). **i** Relative gingival mRNA expression of inflammatory cytokines of *Mettl4*^*−/−*^ and *Mettl4*^*f/f*^ mice that subjected to LO-LIP (*n* = 6). **j**, **l** Representative images of H&E staining (left), TRAP staining (right) and micro-CT (**l**) of the periodontal tissue of *Mettl4*^*−/−*^ and *Mettl4*^*f/f*^ mice that subjected to LO-LIP. The red dash represents the boundary between bone and connective tissue. The yellow dash represents the boundary between connective tissue and epithelium. Scale bars of H&E and TRAP staining, 200 μm. Scale bars of micro-CT, 1 mm. **k** Semi-quantitative analysis of TRAP staining in (**j**) (*n* = 6). **m** Bone loss measurement of differentially treated mouse (*n* = 6). The data are presented as the mean ± SD. ****P < 0.000 1; ***P < 0.001; **P < 0.01. Statistical significance was determined by two-tailed unpaired Student’s *t* test. CLSM confocal laser scanning microscopy, LO low oxygen, NO normal oxygenm PLT platelets, mtDNA mitochondrial DNA, METTL4 methyltransferase-like protein 4, dsDNA double-stranded DNA, cit H3 citrullinated histones H3, TRAP Tartrate resistant acid phosphatase, LIP ligature-induced periodontitis, H&E hematoxylin and eosin, micro-CT micro-computed tomography

To elucidate the role of METTL4 in mtDNA degradation within platelets, platelet METTL4-deficiency mice (*Mettl4*^*f/f*^ *Pf4-Cre*^*+*^*, Mettl4*^*-/-*^) and their littermate control mice (*Mettl4*^*f/f*^ *Pf4-Cre*^*-*^*, Mettl4*^*f/f*^) were used to clarify the role of METTL4 in mtDNA degradation within platelets (Fig. S8a, b). In *Mettl4*^*−/−*^ mice, the protein level of METTL4 in platelets were significantly decreased in platelets compared to that in leukocytes and erythrocytes (Fig. S8c). LO-PLT specimens were obtained from both *Mettl4*^*−/−*^ and *Mettl4*^*f/f*^ mice for evaluating the cytosolic DNA concentration. There was a significant reduction in cytosolic DNA concentration in LO-PLT from *Mettl4*^*−/−*^ mice (Fig. 7a). Flow cytometry analysis demonstrated a high percentage of neutrophil-platelet aggregation in the blood of LO-LIP mice (Fig. 7b, c). Additionally, the formation of NETs was also reduced in neutrophils co-cultured with LO-PLT from *Mettl4*^*−/−*^ mice (Fig. 7d, e). These findings prompted further investigation in whether METTL4 is a potential target for mitigating periodontitis exacerbation under hypoxic conditions.

*Mettl4*^*−/−*^ mice and *Mettl4*^*f/f*^ mice were subsequently subjected to LIP and raised in hypoxic conditions (Fig. 7f). In the *Mettl4*^*−/−*^ mice, NET formation was significantly inhibited in the gingival tissue (Fig. 7g, h). This was accompanied by downregulated expression of inflammatory genes in the gingiva (Fig. 7i). In line with these results, the *Mettl4*^*−/−*^ mice exhibited alleviated symptom of periodontitis (Fig. 7j–m). These findings demonstrate that platelet METTL4 deficiency mitigates hypoxia-driven periodontitis exacerbation.

## Discussion and conclusion

Different clinical conditions that result in hypoxemia have been associated with exacerbation of mucosal inflammation.^35,36^ However, the mechanisms by which hypoxemia aggravates mucosal inflammation remain obscure. The present study identified abnormal platelet activation as a key factor in the worsening of periodontitis under hypoxic conditions. Specifically, activated platelets in gingival tissue promote NETosis, thereby exacerbating periodontitis in hypoxia. Notably, the function of hypoxia-inducible factor-1 in nucleated cells does not explain the hypoxia-induced alterations observed in anucleate platelets.

**Fig. 8:**
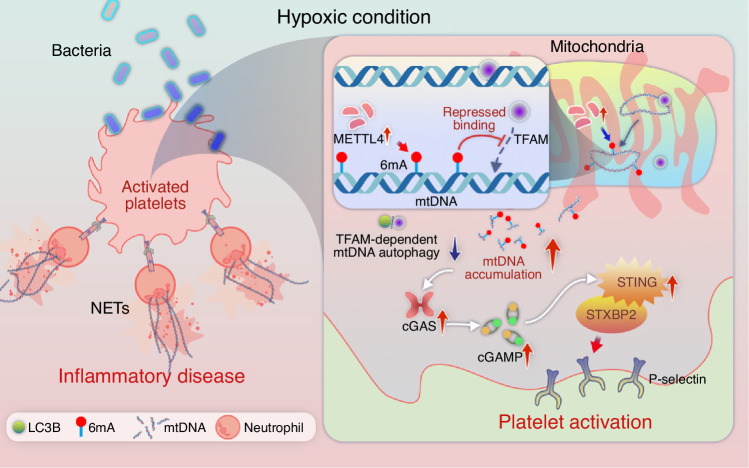
Schematic depiction of platelet-mediated exacerbation of periodontitis under hypoxia. Hypoxia induces METTL4 overexpression in platelets, which mediates 6mA methylation on mtDNA. This modification impairs TFAM-dependent degradation of mtDNA, leading to cytosolic accumulation. Excess mtDNA activates the cGAS-STING pathway, driving platelet hyperactivation, neutrophil-platelet aggregation, and NETs formation, ultimately exacerbating periodontitis

Further investigation revealed that hypoxia activates the cGAS-STING pathway in platelets due to an increased cytosolic mtDNA concentration. This accumulation was driven by the overexpression of platelet METTL4 under hypoxic conditions, which enhanced mtDNA 6 mA methylation and inhibited TFAM-dependent cytosolic mtDNA degradation. Platelet-specific deletion of METTL4 reduced excess mtDNA 6 mA, restored cytosolic mtDNA degradation, suppressed platelet-mediated NETosis, and alleviated periodontitis in hypoxia. These findings suggest that targeting platelet METTL4 may represent a novel therapeutic approach for preventing hypoxemia-related oral mucosal immunopathology (Fig. 8).

Prior studies have primarily focused on the effects of hypoxia-induced alterations in the microbiome on the progression of mucosal inflammation.^17,37^ However, the present study found no significant differences in the biomass or composition of the oral microbiome as periodontitis symptoms worsened under hypoxia. This discrepancy may be attributed to inadequate control of confounding factors in prior studies, such as reduced self-cleaning ability due to xerostomia at high altitudes.

A recent study that accounted for such confounding variables, including reduced self-cleaning ability, investigated the relationship between obstructive sleep apnea and periodontitis.^35^ The findings suggested that low oxygen saturation, rather than external factors, contributes to the prevalence and severity of periodontitis. Results from the present study support this conclusion and further suggest that low oxygen saturation does not affect oxygen tension within periodontal pockets where the oral microbiome resides. Moreover, oxygen levels in periodontal pockets in common forms of periodontitis typically range from 0.7% to 3.8%.^19^ Given these already low levels, it is unlikely that systemic hypoxemia will further reduce oxygen availability in this microenvironment.

The present study revealed dysfunction in mtDNA metabolism within platelets under hypoxic conditions. This phenomenon may not be limited to oral mucosal inflammation. Altered platelet mtDNA metabolism may also be implicated in other hypoxia-related diseases such as pulmonary edema and thromboembolic disorders.^14–16^ However, whether platelets contribute similarly to other forms of mucosal inflammation under hypoxia remains unclear.

Interestingly, colitis symptoms under hypoxic conditions were alleviated by platelet transfusion (Fig. S9). These findings suggest that the effects of hypoxia on platelets may represent a common mechanism underlying hypoxia-related mucosal inflammation. Consequently, targeting platelet METTL4 offers new insights into preventing other inflammatory diseases mediated by hypoxemia. Nevertheless, no METTL4 inhibitors have been developed to date. The functions of METTL4 in eukaryotes also remain poorly understood. Further research is needed to explore its broader implications in hypoxia-related pathological conditions.

Although these findings provide new therapeutic insights, several study limitations warrant discussion. This research focused on platelet alterations and the role of METTL4 overexpression in driving these changes. However, the mechanisms by which hypoxia induces METTL4 overexpression had not been fully explored. Previous studies have shown that hypoxia upregulates METTL4 expression in cardiomyocytes via p53.^34^ However, this mechanism was not confirmed in megakaryocytes in the present study. Further experiments are needed to validate this pathway in megakaryocytes and investigate potential alternative mechanisms.

In summary, this study identifies a mechanism by which platelet activation exacerbates oral mucosal inflammation under hypoxia. METTL4 regulates mtDNA metabolism in platelets, resulting in platelet activation, neutrophil-platelet aggregation, and NET release. Targeting METTL4 and its downstream pathways presents a potential strategy for managing periodontitis. However, additional research is required to determine whether METTL4 inhibition is effective in preventing other forms of mucosal inflammation associated with hypoxia, as current evidence remains insufficient to support this hypothesis.

## Methods

### Ethics approval and consent to participate

Gingival crevicular fluid samples were collected from human with informed consent. Human sample collection was approved by the ethical commission of the Fourth Military Medical University (KQ-YJ-2024-150).

The conduction of all animal experiments was conducted in full compliance with institutional guidelines on animal welfare and received approval from the Medical Ethics Committee of the Fourth Military Medical University (IACUC-2023027).

### Human study population and sample preparation

Based on detailed medical history, all participants were confirmed to be in overall health. Inclusion criteria for the study were: ≥18 years and ≤ 50 years old, generally healthy and diagnosis of periodontitis in stage II according to 2018 international classification of periodontitis. Specific inclusion criteria for the low oxygen periodontitis group included residency in a hypobaric environment with oxygen pressure less than 15 kPa for more than 3 months but less than 1 year. For the normal oxygen periodontitis group, participants must have resided in a normoxic environment (oxygen pressure equals to 21.15 kPa) for at least 5 years. The exclusion criteria that aimed at eliminating confounding variables were as follows: individuals with a history of hepatitis B or C, human immunodeficiency virus infections, previous radiation treatment on head or neck, active cancers, undergone systemic chemotherapy or radiation therapy in the past five years, pregnant or breastfeeding women, diagnoses of diabetes with an HbA1C level exceeding 6%, fewer than three hospital admissions in the last three years, and autoimmune illnesses such as systemic lupus erythematosus and rheumatoid arthritis. Furthermore, additional exclusion criteria encompassed the use of any of the following substances within three months prior to study participation: intaken of systemic antibiotics, any type of corticosteroids, immunosuppressants, cytokine treatments, methotrexate or other immunosuppressive chemotherapeutic agents, as well as high doses of commercial probiotics. Furthermore, the use of tobacco products, including e-cigarettes, within one year prior to screening was also excluded. After screening, 20 patients in LO and 20 patients in NO environments had been included. Gingival crevicular fluid samples were collected from periodontitis patients using Periopaper GCF collection strips (Oraflow, USA). Samples were obtained from four specific sites on the left maxillary first molar: the distal buccal, distal lingual, mesial buccal, and mesial lingual sites. In cases where the left maxillary first molar was lost or free of inflammation, the collection prioritization proceeded as follows: first, the first molar of the right maxilla; second, the first molar of the left mandible; and finally, the first molar of the right mandible. And the samples were subsequently stocked in −80 °C. 100 µL phosphate-buffered saline (PBS) was used for extraction from periopaper obtained from every patient and extracted GCF was then subjected to use.

### Animals

All studies used C57BL/6J littermate controls. The Experimental animal center of Fourth Military Medical University provided the wild-type mice. Rosa26iDTR mice were breed with platelet factor 4 (PF4)-cre mice to obtain PF4-diphtheria toxin receptor (DTR) mice. *Mettl4*^*f/f*^ mice were subsequently hybridized with PF4-Cre mice to achieve methyltransferase-like protein 4 (METTL4) deletion in megakaryocytes/platelets. All mice were subjected to use at 6 weeks of age without sex bias. Each group has 6 biological replicates. The sources of genotyped mice and the primer sequences for identifying genotypes were provided in Table S1.

### Ligature-induced periodontitis (LIP) and hypobaric hypoxia simulation

Mice were anesthetized with pentobarbital sodium (50 μg/g). Both side of maxillary second molar tooth was surrounded by a silk ligature (5-0) and examination on detachment of ligature were conducted every 3 days. The ligature was removed 14 days later. For hypobaric hypoxia simulation, mice were placed in a decompression chamber (Hongyuan, China) and exposed to hypobaric hypoxia at an oxygen pressure of 12.32 kPa, as previous described.^37^ The chamber conditions were maintained at (25 ± 5) °C and 50% ± 5% humidity, respectively. Airflow in the chamber was set at 3 L/min. The animals were provided food and water inside the chamber.

### Micro-CT

Mouse mandibles were scanned to evaluate bone loss by Inveon micro-CT system (Siemens, Germany). 3D reconstruction of mouse mandible was generated with an 8 μm spatial resolution. And alveolar bone loss was measure as previously mentioned.^8^

### Antibiotics and DNase I administration

Antibiotics (minocycline hydrochloride, 10 mg/mL; metronidazole, 200 mg/mL) or DNase I (125 mg/mL) were resolved in 0.9% saline and stored in 4 °C until use. The silk ligature was soaked in antibiotic suspention, DNase I suspension or 0.9% saline for 24 h at 4 °C before using for LIP. These silk ligatures were updated daily during the LIP procedure.

### 16S rRNA gene sequencing of oral microbiome

Microbial samples were obtained from mouse oral cavity by ligature placed around maxillary second molar tooth. Primers 341 F and 806 R were used to amplify the 16S rDNA V3-V4 region. DNA extraction and purification, raw data acquire and analysis was performed by Gene Denovo.

### Ribonucleic acid sequencing

Mucogingival tissue strips harvested from the buccal and lingual sides, spanning from the first to the third molar (*n* = 3) were quick-freezed by liquid nitrogen and send to Novogene for total RNA extraction. And Novogene also generated RNA sequencing data from the extracted RNA and analyze the raw data was processed as previously described.^38^

### In vitro simulation of periodontitis microenvironment

*Porphyromonas gingivalis* (*Pg*, ATCC 33277) were suspended in RPMI 1640 medium (Gibco, USA) (100 µg/mL lysozyme) at a concentration of 10^7^ per mL. This medium was then frozen at −20 °C and melt at 4 °C for 3 cycles and filtered by 0.22 µm syringe filter. This medium was co cultured with platelets or neutrophils for in vitro simulation of periodontitis microenvitronmets.

### Flow cytometry

For analyzing the immune cells in mouse gingivae, mouse gingivae tissues were processed into single cell suspension as as previously described.^39^ For analyzing neutrophil-platelet aggregation, mouse blood was treated by erythrocyte lysis buffer (KHCO_3_, 10 mol/L; NH_4_Cl, 159 mol/L; Na_2_EDTA, 0.1 mol/L, pH 7.2-7.4) for 15 min at room temperature. For platelets activation analysis, mouse whole blood was directly incubated with Pg medium and antibodies.

Flow cytometric analysis was performed on BD FACSCelesta. Propidium iodide (5 µg/mL, Solarbio, China) were used to separate live and dead cell for 488 nm excitation. Anti-FcγIII/I receptor (1:200, Biolegend, USA) were used for block. And then primary antibodies against surface markers were incubated in cell staining buffer (PBS with 2% fetal bovine serum) for 30 min at 4°C. The monoclonal antibodies used for cell surface phenotypic were listed in Table S1. Data analysis was performed by Flowjo software.

### Cells preparation and incubation

Mouse neutrophils and platelets were extracted from mouse blood (sodium citrate; 0.38%) via commercial kits (Solarbio, China, listed in Table S1). Cell viability and purity were assessed by flow cytometry.

### Polymerase chain reaction (PCR)

The cytosolic DNA or whole DNA in platelets were extracted by TIANamp Genomic DNA Kit (TIANGEN, China) and amplified using Ex Premier™ DNA Polymerase Dye plus (Takara, Japan), containing 100 nmol/L of the primer metioned in Table S1. Amplified products were analyzed on 1% agarose gels and GoldView Nucleic acid gel stain (Accurate Biology, China) were used for staining DNA. Platelets cytosolic DNA were conducted to PCR to identify its source. And the integrity of platelet mtDNA were also evaluated by PCR.

### Enzyme-linked immunosorbent assay (ELISA)

Commercial ELISA kits were used to assess the concentration of interleukin-1β, Prostaglandin E2, neutrophil elastase, myeloperoxidase-DNA complex and cGAMP according to instructions. GCF samples were prepared as mentioned before. The mouse gingivae were quick-frozen by liquid nitrogen after harvested. And the frozen tissues were smashed into powder for total protein extraction. Cell samples such as neutrophils and platelets were collected and subjected for ELISA detection after undergoing indicated treatment.

### Scanning electron microscopy

Neutrophils and platelets were seed on cell climbing sheets (14 mm in diameter) to undergo the indicated treatment. Specimens were then fixed by 2.5% glutaraldehyde in 4 °C for 30 min and washed by PBS before dehydrated by ascending ethanol series for 2 min each time and 100% ethanol were used for twice. Hexamethyldisilane was then drop on specimen, which was then air-dried for 2 h. The samples were subsequently fixed on sample holders by carbon tape and sputter-coated with gold/palladium. The examination of samples was conducted by scanning electron microscope (FE-SEM, S-4800, Hitachi, Japan).

### Depletion and transfusion of neutrophils and platelets

RNase-free water was used as solvent for diphtheria toxin. Diphtheria toxin (DT) solution was stocked in -80° C until use. Recipients were transfused with DT (20 ng/g body weight) that diluted in PBS via intraperitoneally injection daily. Harvested neutrophils and platelets from donors were resuspended in PBS. Recipients were transfused with 200 μL of neutrophil suspension (5 × 10^6^ cells per mL) or platelets suspention (2 × 10^9^ cells per mL) via tail-vein injection every 2 days.

### Measurement of cytosolic mtDNA and mtDNA in mitochondrion

Platelets were stimulated as indicated. Mitochondrion and cytosol were isolated by Mitochondria Isolation Kit (MCE, USA). qPCR was performed as previously mentioned, from both cytosolic fractions and mitochondria using mtDNA primers. The relative primers were exhibited in Table S1. A standard curve for each gene were created by mtDNA genome standard samples with known copy numbers and were used to access the copy number of the target gene in the samples.

### Immunoprecipitation

Platelets protein was extracted as mentioned before. Protein A/G magnetic beads were incubated with primary antibodies or control IgG at 4 °C overnight. Platelet lysis was then incubated with treated beads at 4 °C overnight. The beads were then separated and acid elution buffer were then used for separate beads and indicated protein. The combination of STING to STXBP2 in platelets were assessed by immunoprecipitation.

### STING palmitoylation detection

The assay was performed by IP-ABE Palmitoylation Kit (AIMS, China) according to the manufacturer’s instructions. Experimental principle is accessible as previously studied.^40^

### Chromatin immunoprecipitation (ChIP)

The BeyoChIP™ ChIP ChIPAssay Kit (Beyotime, China) were used to conduct ChIP assay. The mtDNA was purified and real-time qPCR was used to determine the presence of TFAM at the mtDNA promoter regions, including HSP1, HSP2, and LSP. The relative primers are listed in Table S1. ChIP assay were adopted to assess the combination of TFAM to mtDNA in platelets.

### Statistical analyses

Statistical analysis was conducted by GraphPad Prism v8. And all quantitative data were displayed in means ± standard deviations. One-way analysis of variance (ANOVA) was used for analyze multiple comparation among groups. Two-tailed unpaired Student’s *t* test was adopted to evaluate differences between two groups. Two-way ANOVA was used to in CHIP analysis.

## Supplementary information

Supplementary Material

## Data Availability

All the data that supported the finding in this study are presented in this article and materials. Any additional data that required to reanalyze is accessible from the contact upon reasonable request (niulina831013@126.com).
