# Supplementary material for: Platelet methyltransferase-like protein 4-mediated mitochondrial DNA metabolic disorder exacerbates oral mucosal immunopathology in hypoxia
Source: Int J Oral Sci. 2025 Jun 12;17:49. doi: 10.1038/s41368-025-00373-9 (PMC12163075; doi:10.1038/s41368-025-00373-9)
Supplement: Supplementary file 1 — Supplementary Material [file 41368_2025_373_MOESM1_ESM.docx]

**Platelet METTL4-mediated mtDNA metabolic disorder exacerbates oral mucosal immunopathology in hypoxia**

Yi-na Zhu^†,1^, Mei-chen Wan^†,1^, Yu-tong Fu^†,1^, Jun-ting Gu^1^, Zhao-yang Ren^1^, Yun Wang^1^, Ke-hui Xu^1^, Jing Li^1^, Man-jiang Xie^2^, Kai Jiao^3^, Franklin Tay^4^, Li-na Niu^1,*^

^†^ Equal contributors

^1^ State Key Laboratory of Oral and Maxillofacial Reconstruction and Regeneration, National Clinical Research Center for Oral Diseases, Shaanxi Key, Laboratory of Stomatology, Department of Prosthodontics, School of Stomatology, The Fourth Military Medical University, Xi’an, Shaanxi, 710032, China

^2^ Department of Aerospace Physiology, Fourth Military Medical University, Xi'an, Shaanxi, 710032, China

^3^ Department of Stomatology, Tangdu Hospital, The Fourth Military Medical University, Xi’an, 710000, China

^4^ The Dental College of Georgia, Augusta University, Augusta, GA, 30912, USA

**Email address:** Yi-na Zhu (870863503@qq.com), Mei-chen Wan (wmc1125@163.com), Yu-tong Fu (1355080530@qq.com), Jun-ting Gu (15094031504@163.com), Zhao-yang Ren (renzy1013@163.com), Yun Wang (852600641@qq.com), Ke-hui Xu (2744404972@qq.com), Jing Li (769266996@qq.com), Man-jiang Xie (xiemanjiang@fmmu.edu.cn), Kai Jiao (kjiao1@163.com), Franklin Tay (tayfranklin7@gmail.com), Li-na Niu ([niulina831013@126.com](mailto:niulina831013@126.com)).

***Corresponding author**: Dr. Li-na Niu, The Fourth Military Medical University, Xi’an, Shaanxi, China. Email: [niulina831013@126.com](mailto:niulina831013@126.com). Telephone: 029-84776001

**Supplementary Information**

**Supplementary Materials and Methods**

**Fig. S1** Contribution of oral microbiome to periodontitis exacerbated under hypoxic condition.

**Fig. S2** Gating strategy for flow cytometry analysis of immune cell populations

**Fig. S3** Effects of DNase I on periodontitis under hypoxic condition.

**Fig. S4** Effects of oral microbiome on NETs formation *in vitro*.

**Fig. S5** Effects of neutrophils on NETs formation and periodontitis under hypoxic condition.

**Fig. S6** The level of mtROS and mtDNA integrity in activated platelets.

**Fig. S7** The effect of hypoxia on the protein level of TREX1 and TFAM in platelets.

**Fig. S8** Establishment of platelet-specific *Mettl4* gene deletion mouse models.

**Fig. S9** Effects of platelet transfusion on colitis at hypoxic condition.

**Table S1** Major Resource Table

**Supplementary Materials and Methods**

*Histological analysis*

Mouse mandibles were harvested and then fixed in 4% paraformaldehyde for 1 days in 4°C. The tissues were subsequently decalcified in 20% ethylene diamine tetraacetic acid and embedded in paraffin. The thickness of tissue specimen for hematoxylin and eosin staining (Solarbio, China) or tartrate-resistant acid phosphatase staining is 10 mm. The semi-quantitative analysis of tartrate-resistant acid phosphatase staining was performed by ImageJ software.

*Quantitative real-time PCR*

Trizol reagent (Invitrogen, USA), isopropyl alcohol and chloroform were used for RNA extraction, followed by exclamation of genome DNA and reverse transcription via the PrimeScript RT Master Mix with gDNA Eraser (Takara, Japan). The mRNA levels were quantified by the TB Green Premix Ex Taq II kit (Takara, Japan) on the Biorad CFX Connect Real-Time PCR System. Relative primer sequences are listed in **Supplementary Table S1**. Relative gene expression was normalized to β-actin mRNA level. The expression of inflammatory cytokines (*Il6*, *Il23p19*, *Il17* and *rankl*) in mouse gingivae were analysised by qPCR.

*Western blot assay*

Washed with pre-chilled phosphate-buffered saline, cells were subsequently melted in lysis buffer (NCM Biotech, China) contained 10 µL protease and phosphatase inhibitors (NCM Biotech, China) per mL. Protein concentrations were measured by BCA assay kit. If the concentration varies, lysis buffer will be used to dilute samples into equal concentration. Denatured lysates were subjected to 10% or 12.5% SDS-PAGE gel (Epizyme, China) and transferred onto polyvinylidene difluoride membranes (Millipore, USA). Nonfat milk (5%) were used for block membranes at room temperature for 2 h, then the membranes were incubated with primary antibodies (listed in **Supplementary Table S1**) overnight at 4°C. Washed by Tris buffered saline with Tween-20 (TBST), they were subjected to horseradish peroxidase-conjugated secondary antibodies and incubated at room temperature for 2 h. For signal detection of protein, enhanced chemiluminescence substrate (Thermo Fisher Scientific, USA) were used after washed by TBST. The protein level of cit H3 in neutrophils and TREX3, TFAM and METTL4 in platelets were analyzed by western blot.

*Immunofluorescence analysis*

Paraffin sections of mouse periodontal tissue were deparaffinized and antigen retrieval was achieved by antigen retrieval buffer (Boster Biological Technology, China). Cell samples were fixed with 4% paraformaldehyde. After permeabilized by 1% Triton X-100 for tissue specimen and 0.5% Triton X-100 for cell samples, these samples were incubated with goat serum blocking solution (Boster Biological Technology, China) for 30 min at room temperature, which was then removed. And the primary antibodies were incubated with samples at 4 °C overnight. After washing by phosphate-buffered saline, secondary antibodies were added and incubated for 1 h in the dark at room temperature. 4′,6-diamidino-2-phenylindole and Sybr Green I were subsequently incubated for 10 mins in the dark at room temperature if used. The specimens were finally mounted in antifade mounting medium for confocal scanning laser microscopy. The primary and second antibodies used are listed in **Supplementary Table S1**.

Immunofluorescence images were captured by Nikon A1R confocal microscope (Nikon, Japan). Relative fluorescence area was calculated using the ImageJ software (National Institute of Health, Bethesda, MD, USA). In the relative fluorescence area comparison, the average of five views in each sample was used as one data point. 6 different samples were compared per group. Such a method has been used extensively for quantification of immunofluorescent staining to avoid observer bias. The infiltration of neutrophils (Ly6G), the presence of neutrophil extracellular traps (NETs) (cit H3), and the localization of platelets (CD41) in mouse periodontal tissue were assessed using immunofluorescence analysis. Additionally, the formation of NETs (cit H3 and DNA), along with the localization of DNA, TFAM, METTL4, N6- 6mA, as well as the levels of mtROS in platelets, were evaluated by immunofluorescence.

*Dot blotting*

Extracted mtDNA were diluted to 50 ng/uL. 2.5 μL mtDNA suspention were loaded on Hybond N+ membranes (Labselect, China). Membranes were air-dried in 65% for 30 mins and then crosslinked by ultraviolet stratalinker 2400 at 150mJ/cm^2^. Nonfat milk (5%) in TBST was used for blocking and anti-6mA antibody (1: 1000) was incubated with membranes at 4 °C overnight. Horseradish peroxidase-conjugated anti-mouse IgG were incubated for 2 h at room temperature after TBST washed. Enhanced chemiluminescence substrate was used after TBST washed. Dot blotting was used for evaluating the 6mA level of platelet mtDNA.


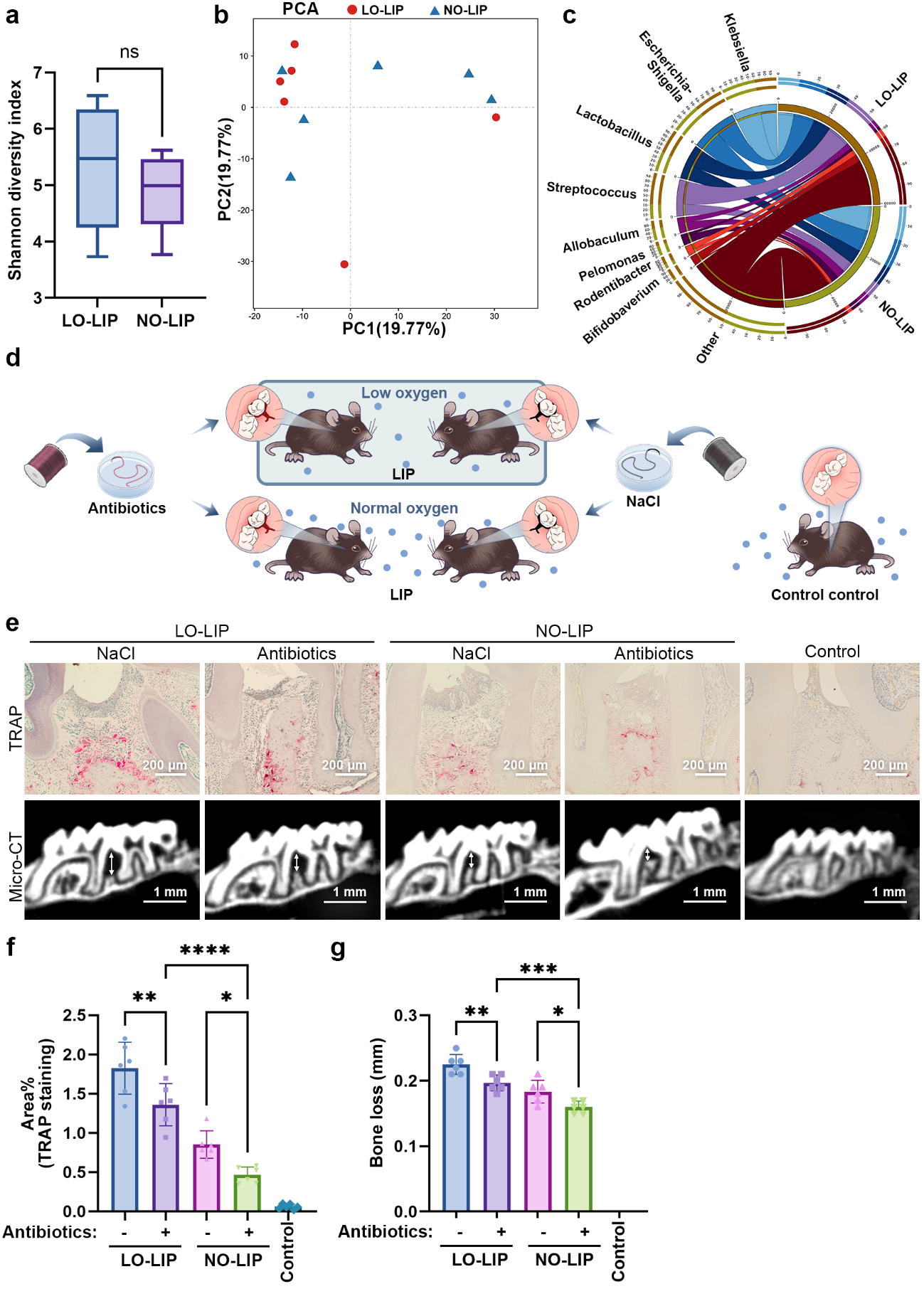


**Fig. S1** Contribution of oral microbiome to periodontitis exacerbated under hypoxic condition. **a,b** 16S rRNA sequencing of microbial diversity measured through the Shannon diversity index (**a**) and principal coordinates analysis (**b**) of oral samples from LO-LIP and NO-LIP mice (*n* = 6 per group). **c** Microbiome composition at the OTU level. The 20 most abundant OTUs depicted are classified at the genus level. **d** Schematic illustration of the experimental design. **e** Representative images of TRAP staining (upwards) and micro-CT (downwards) of periodontal tissues of differentially treated mice. Scale bars of TRAP staining: 200 μm. Scale bars of micro-CT: 1 mm. **f** Semi quantitative analysis of TRAP staining in (**e**) (*n* = 6). **g** Bone loss measurement of differentially treated mice (*n* = 6 per group). The data are presented as the mean ± SD. ***P < 0.001; **P < 0.01; *P < 0.05; ns, no significance. Statistical significance was determined by two-tailed unpaired Student’s t-test (**a**) or one-way ANOVA (**f, g**). (LIP, ligature-induced periodontitis; TRAP, Tartrate resistant acid phosphatase; micro-CT; micro-computed tomography; LO, low oxygen; NO, normal oxygen; OTU, operational taxonomic units, ns, no significance)


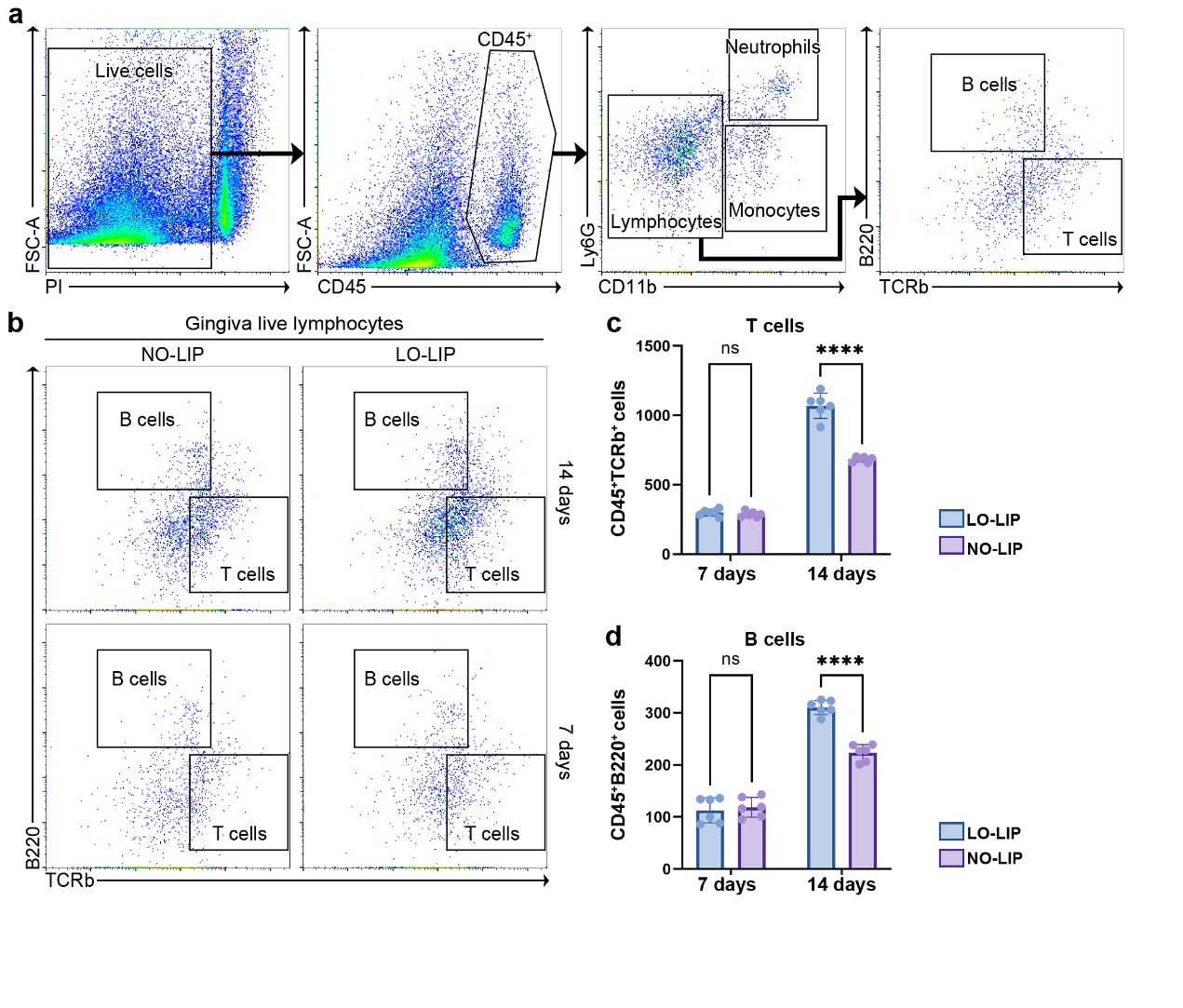


**Fig. S2** Gating strategy for flow cytometry analysis of immune cell populations (related to **Fig. 2**). **a** Single cells were determined by gating for FSC and SSC. Live cells were gated for CD45^+^cells. Neutrophils were defined as CD11b^+^Ly6G^+^ cells. Monocytes-macrophages were defined as CD11b^+^Ly6G^−^ cells. The Ly6G^-^CD11b^-^ populations were further gated for lymphocytes. T cell and B cell populations were determined by the presence of TCRb and B220 markers, respectively. FACS plot (**b**) and counts of T cells (**c**) and B cells (**d**) in gingivae of LO-LIP and NO-LIP mice after subjected to LIP for 7 days and 14 days (*n* = 6 per group). The data are presented as the mean ± SD. ****P < 0.0001; ***P < 0.001; **P < 0.05; ns, no significance. Statistical significance was determined by two-tailed unpaired Student’s t-test. (LIP, ligature-induced periodontitis; LO, low oxygen; NO, normal oxygen; FSC, forward scatter; SSC, side scatter; FACS, fluorescence activated cell sorting)


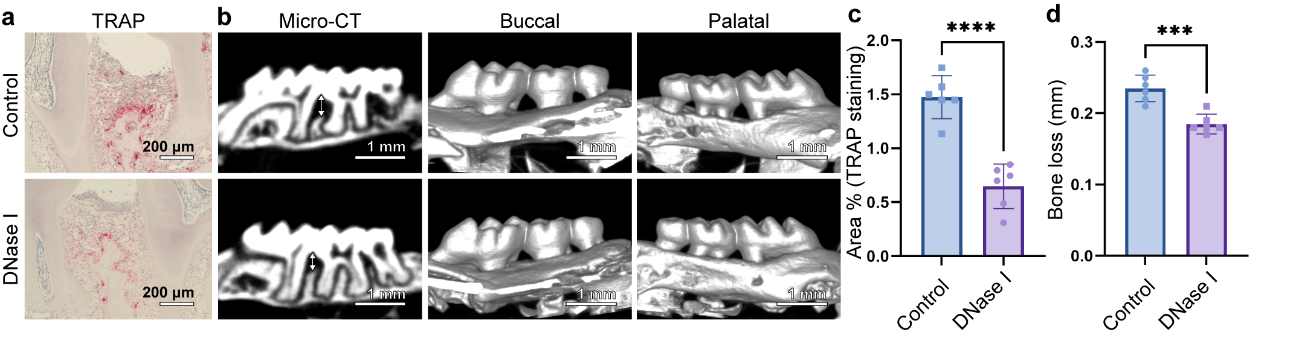


**Fig. S3** Effects of DNase I on periodontitis under hypoxic condition. **a, b**. Representative TRAP staining (**a**) and micro-CT (**b**) of the periodontal tissue of DNase I-treated mice and littermate controls. Scale bars of TRAP staining: 200 μm. Scale bars of micro-CT: 1 mm. **c** Semi quantitative analysis of TRAP staining in (**a**) (*n* = 6). **d** Bone loss measurement of DNase I-treated mice and littermate controls (*n* = 6 per group). The data are presented as the mean ± SD. ***P < 0.001; Statistical significance was determined by two-tailed unpaired Student’s t-test. (TRAP, Tartrate resistant acid phosphatase; micro-CT, micro-computed tomography)


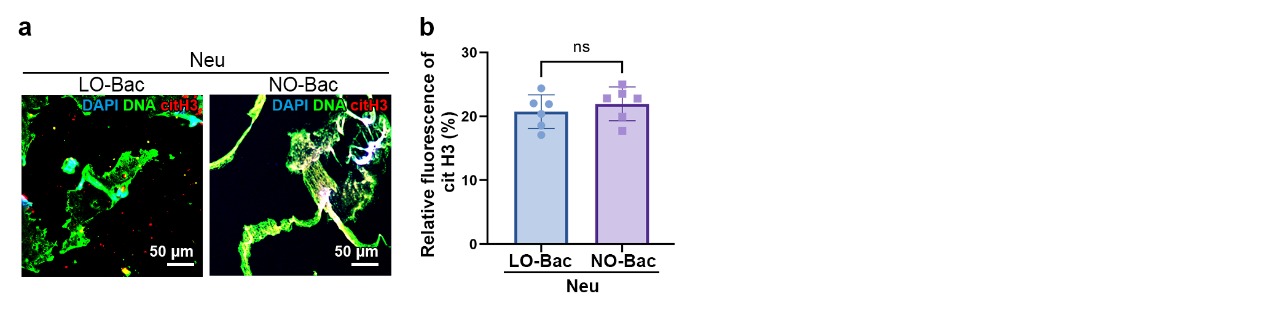


**Fig. S4** Effects of oral microbiome on NETs formation *in vitro*. **a** Representative CLSM images of neutrophils incubated with LO-Bac and NO-Bac. Scale bars: 50 μm. **b** Semi-quantitative analysis of cit H3 signaling in (**c**) (*n* = 6 per group). The data are presented as the mean ± SD. ns, no significance. Statistical significance was determined by two-tailed unpaired Student’s t-test. (Neu, neutrophils; LO, low oxygen; NO, normal oxygen; Bac, bacterial; CLSM, confocal laser scanning microscopy; cit H3, citrullinated histone H3).


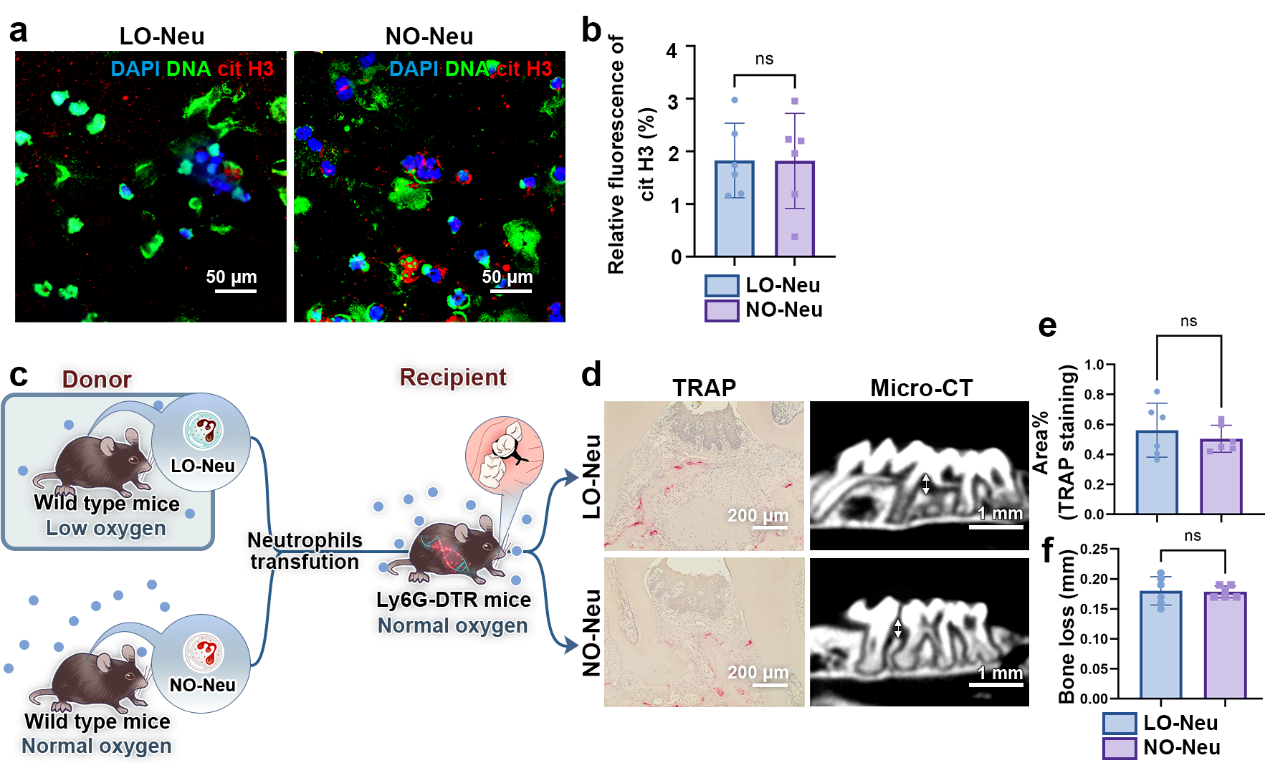


**Fig. S5** Effects of neutrophils on NETs formation and periodontitis under hypoxic condition. **a** Representative CLSM images of LO-Neu and NO-Neu. Scale bars: 50 μm. **b** Semi-quantitative analysis of cit H3 signaling in (**a**) (*n* = 6 per group). **c** Schematical design of the *in vivo* experiments. **d** Representative TRAP staining (left) and micro-CT (right) images of the periodontal tissues of Ly6G-DTR mice transfused with LO-Neu and NO-Neu. Scale bars of TRAP staining: 200 μm. Scale bars of micro-CT: 1 mm. **e** Semi quantitative analysis of TRAP staining in (**d**) (*n* = 6). **f** Bone loss measurement of differently treated Ly6G-DTR mice (*n* = 6 per group). The data are presented as the mean ± SD. ns, no significance. Statistical significance was determined by two-tailed unpaired Student’s t-test. (Neu, neutrophils; LO, low oxygen; NO, normal oxygen; CLSM, confocal laser scanning microscopy; cit H3, citrullinated histone H3)

**
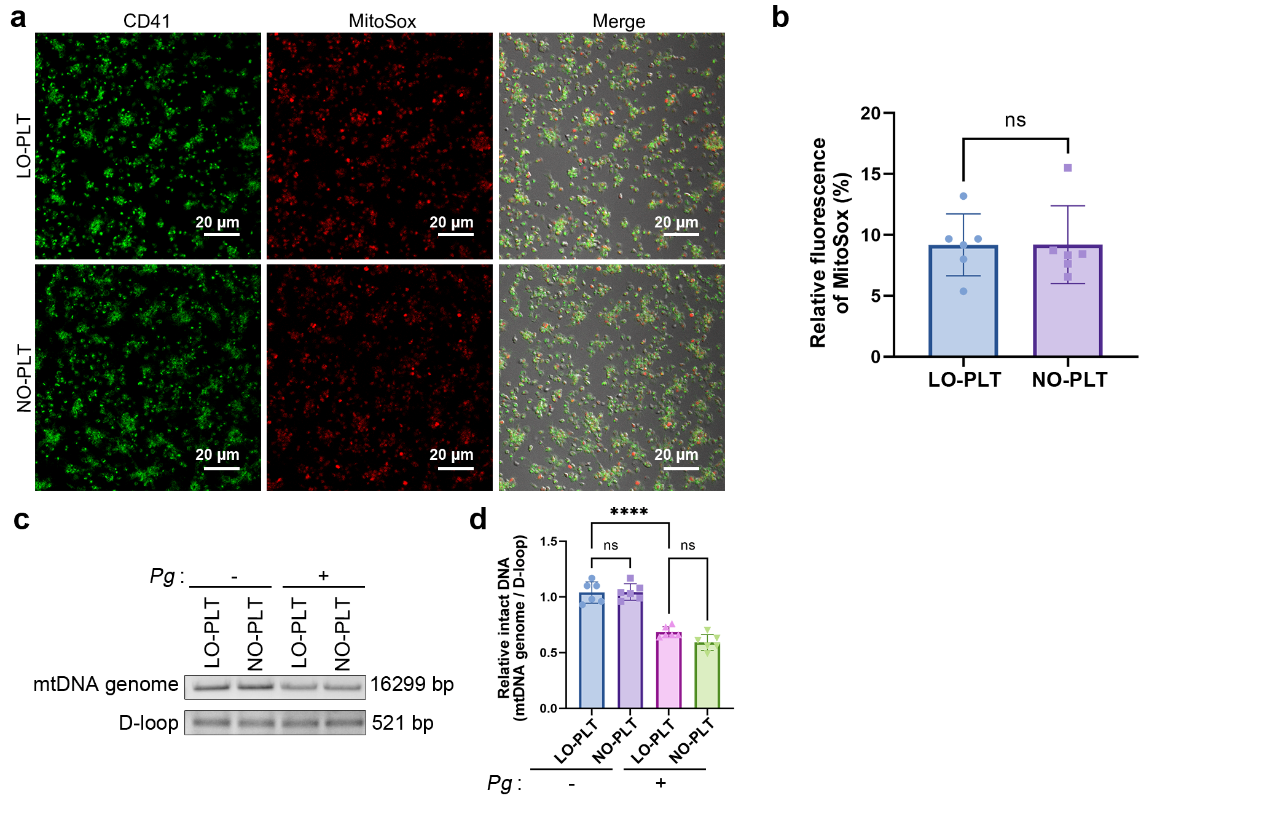
**

**Fig. S6** The level of mtROS and mtDNA integrity in activated platelets. **a** CLSM images of LO-PLT and NO-PLT incubated in *Pg* medium, stained with CD41 (green) and MitoSox (red). Scale bar: 20 μm. **b** Semi-quantitative of the MitoSox signaling in (**a**) (*n* = 6 per group). **c** mtDNA PCR amplification products (entire mitochondrial genome, 16,299 bp, and D-loop region, 591 bp) from HO-PLT and LO-PLT incubated in *Pg* medium or not. **d** Semi-quantitative analysis in (**c**). Relative intact mtDNA measured by the ratio of whole mtDNA genome to D-loop region in the PLTs (*n* = 6 per group). The data are presented as the mean ± SD. ****P < 0.0001; ***P < 0.001; **P < 0.05; ns, no significance. Statistical significance was determined by two-tailed unpaired Student’s t-test (**b**) or one-way ANOVA (**d**). (CLSM, confocal laser scanning microscopy; LO, low oxygen; NO, normal oxygen; PLT, platelets)


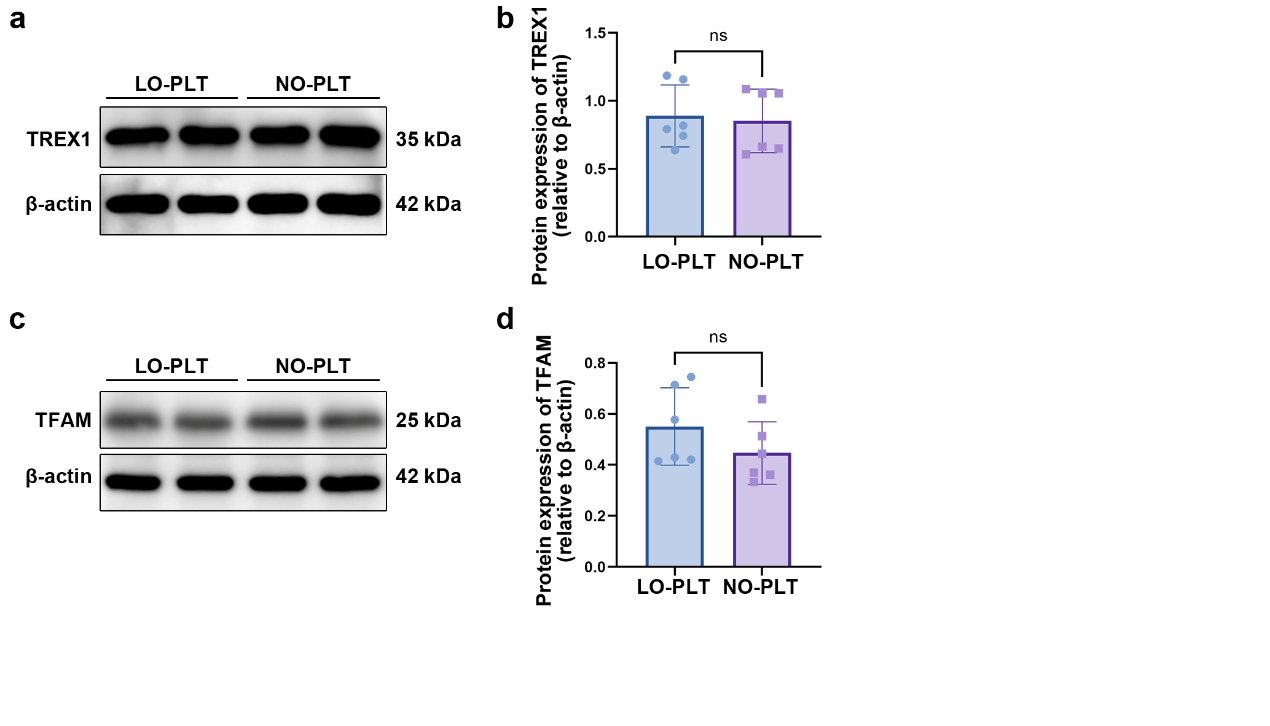


**Fig. S7** The effect of hypoxia on the protein level of TREX1 and TFAM in platelets. **a** The expression of TREX in LO-PLT and NO-PLT. **b** Semi-quantitative analysis of TREX in (**a**) (*n* = 6 per group). **c** The expression of TFAM in LO-PLT and NO-PLT. **d** Semi-quantitative analysis of TREX in (**c**) (*n* = 6 per group). The data are presented as the mean ± SD. ns, no significance. Statistical significance was determined by two-tailed unpaired Student’s t-test. (TREX1, three prime repair exonuclease 1; TFAM, mitochondrial transcriptional factor A; LO, low oxygen; NO, normal oxygen; PLT, platelets)

**
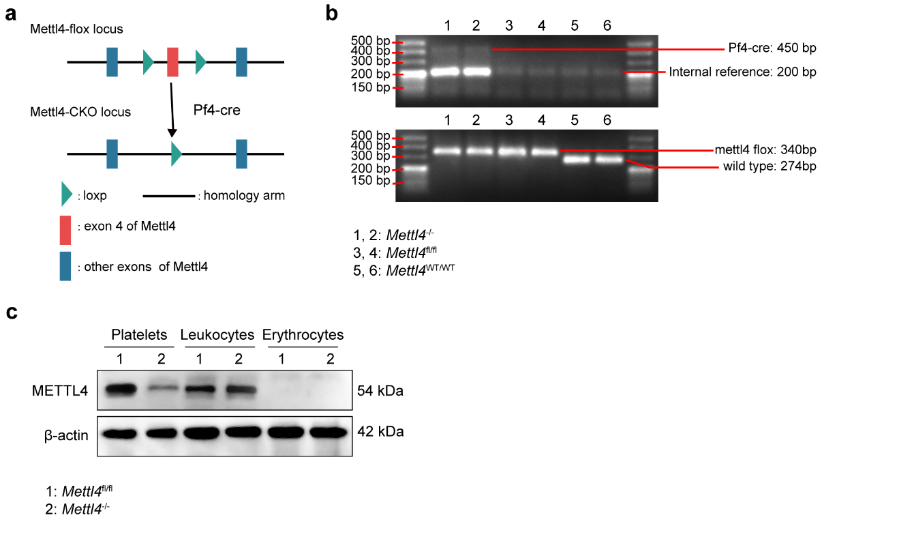
**

**Fig. S8** Establishment of platelet-specific *Mettl4* gene deletion mouse models. **a** The construction strategy of METTL4 conditional knockout mice. *Mettl4* floxed (*Mettl4*^fl/fl^) mouse models were established by inserting loxP sequences on both sides of exon 4 of the *Mettl4* gene. **b** Genotyping of *Mettl4*^-/-^ mouse lines by PCR. **c** Protein expression level of METTL4 in platelets, leukocytes and erythrocytes of *Mettl4*^-/-^ and *Mettl4*^fl/fl^ mice. (METTL4, methyltransferase-like protein 4)

**
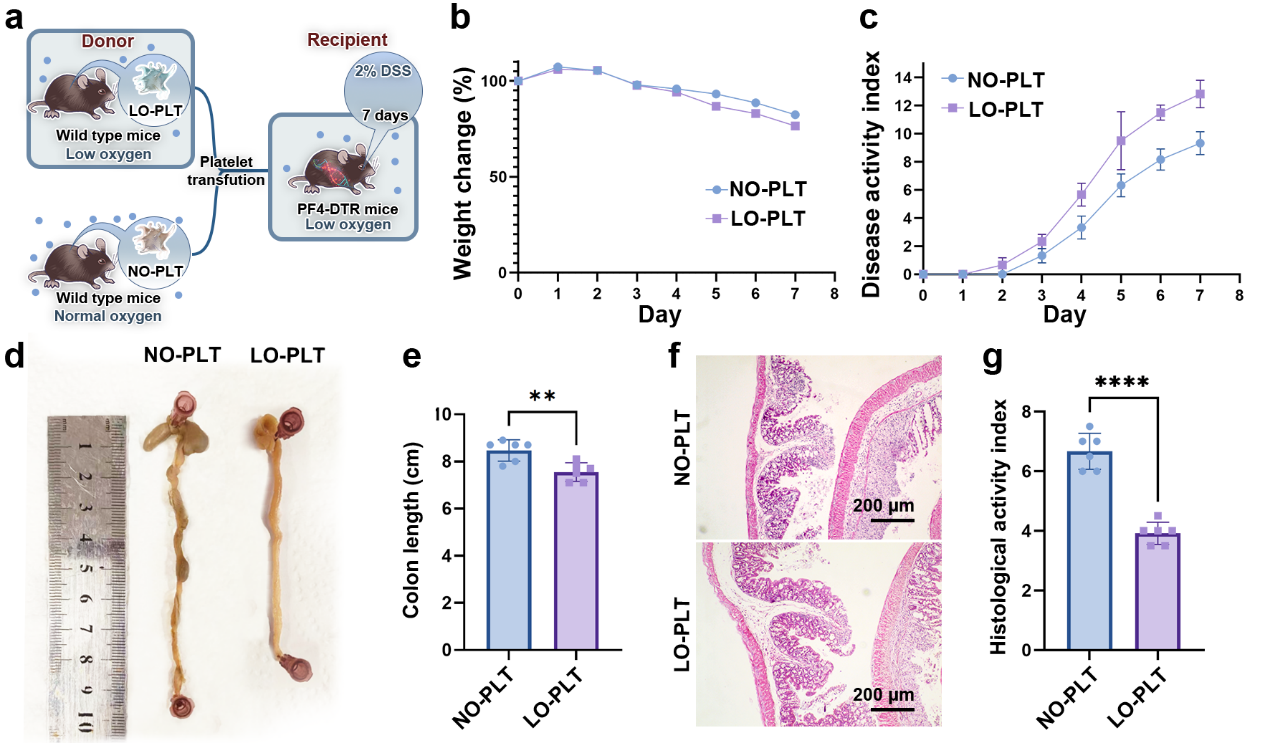
**

**Fig. S9** Effects of platelet transfusion on colitis at hypoxic condition. **a** Schematic design of the *in vivo* experiments. A DSS-induced colitis model was established on PF4-DTR mice at LO-environment. DT were injected daily to deplete host platelets. LO-PLT or NO-PLT were subsequently transfused. Mice were sacrificed after subjected to DSS for 7 days. **b** Body weight of mice transfused with LO-PLT or NO-PLT during DSS-induced colitis (*n* = 6 per group). **c–g** Disease activity index (**c**), colon length (**d, e**), and histological score (**f, g**) of mice transfused with LO-PLT or NO-PLT after DSS induction. Scale bars: 200 μm. (*n* = 6 per group). The data are presented as the mean ± SD. ****P < 0.0001; **P < 0.05. Statistical significance was determined by two-tailed unpaired Student’s t-test (DSS; dextran sodium sulfate; LO, low oxygen; NO, normal oxygen; PLT, platelets; PF4, platelet factor 4)

**Table S1.** Major Resource Table

| REAGENT or RESOURCE | Source | Identifier |
| --- | --- | --- |
| Animal | | |
| C57BL/6 mice | The Fourth Military Medical University's Laboratory Animal Research Center, China | N/A |
| *Padi4*^-/-^ mice | Shanghai Model organisms, China | NM-KO-190334 |
| Ly6G-DTR mice | Kindly gift from Prof. Yin-ming Liang | N/A |
| *Platelet factor 4 (Pf4)-Cre* mice | Model animal research center of Nanjing University, China | N/A |
| Rosa26iDTR mice | Jackson laboratory, USA | 007900 |
| *Mettl4^f/f^* mice | Shanghai Model organisms, China | NM-CKO-220279 |
| Antibodies | | |
| Rabbit Anti-Citrullinated histone H3 | Abcam, USA | ab5103 |
| Rabbit Anti-TFAM | Proteintech, China | 22586-1-AP |
| Rabbit Anti-METTL4 | Abclonal, China | A9294 |
| Mouse Anti-dsDNA | Abcam, USA | ab27156 |
| Rabbit Anti-STING | Proteintech, China | 19851-1-AP |
| Mouse Anti-STXBP2 | Proteintech, China | 66238-1-lg |
| Rabbit Anti-Ly6G | Abcam, USA | ab238132 |
| Mouse Anti-TOM20 | Proteintech, China | 66777-1-lg |
| Rat Anti-mouse CD11b (APC) | Biolegend, USA | 101211 |
| TruStain fcX™ PLUS (anti-mouse CD16/32) Antibody | Biolegend, USA | 156603 |
| Rat Anti-mouse CD45 (FITC) | Biolegend, USA | 103107 |
| Rat Anti-mouse Ly6G (Brilliant Violet™ 605) | Biolegend, USA | 127639 |
| Rat Anti-mouse TCR β (Alexa Fluor® 700) | Biolegend, USA | 109223 |
| Rat Anti-mouse B220 (PerCP/Cyanine5.5) | Biolegend, USA | 103235 |
| Mouse Anti-mouse CD62P (PE) | Biolegend, USA | 148305 |
| Rat Anti-mouse CD41 Antibody (FITC) | Biolegend, USA | 133903 |
| Goat Anti-Rabbit IgG H&L (Alexa Fluor® 405) | Abcam, USA | ab175652 |
| Goat Anti-Rabbit IgG H&L (Alexa Fluor® 647) | Abcam, USA | ab150079 |
| Goat Anti-Mouse IgG H&L (Alexa Fluor® 647) | Abcam, USA | ab150115 |
| Goat Anti-Mouse IgG H&L (Alexa Fluor® 488) | Abcam, USA | ab150113 |
| Rabbit Anti-phospho-STING/TMEM173-S366 | Abclonal, China | AP1223 |
| Rabbit Anti-β-actin | Engibody, USA | AT0009 |
| HRP-conjugated Affinipure Goat Anti- Mouse IgG (H+L) | Proteintech, China | SA00001-1 |
| HRP-conjugated Affinipure Goat Anti-Rabbit IgG (H+L) | Proteintech, China | SA00001-2 |
| Chemicals, peptides, and recombinant proteins | | |
| 4',6-diamidino-2-phenylindole | Sigma, USA | 28718-90-3 |
| DNase I | Solarbio, China | 9003-98-9 |
| MitoSox Red | MCE, USA | HY-D1055 |
| RPMI 1640 medium | Gibco, USA | 11875119 |
| Collagenase, Type IV | Gibco, USA | 17018029 |
| Protease and Phosphatase Inhibitor Cocktail | NCM Biotech, China | P002 |
| Fetal Bovine Serum | Pricella, China | 164210-50 |
| Triton X-100 | Solarbio, China | T8200 |
| Penicillin streptomycin | Pricella, China | PB180120 |
| TRIzol™ | Invitrogen, USA | 15596018CN |
| 4% Paraformaldehyde Fix Solution | Beyotime, China | P0099 |
| SYBR Green I | Yeasen, China | 10222ES60 |
| Propidium Iodide | Solarbio, China | P8080 |
| Antigen Retrieval Buffer | Boster Biological Technology, China | AR0026 |
| Goat Serum Blocking Solution | Boster Biological Technology, China | AR0009 |
| Minocycline hydrochloride | Solarbio, China | IM0450 |
| Metronidazole | Solarbio, China | IM0230 |
| C-ST5 peptide: GRKKRRQRRRQQTGDHAGIKD | Abclonal, China | N/A |
| Critical commercial assays | | |
| Pierce BCA Protein Assay | Thermo Scientific, USA | 23225 |
| TB GreenÒ Premix Ex TaqÔII (Tli RNaseH Plus) | Takara, Japan | RR820A |
| Mouse peripheral blood neutrophil isolation kit | Solarbio, China | P9201 |
| Mouse peripheral blood platelet isolation kit | Solarbio, China | P1620 |
| Human IL-1β (Interleukin 1 Beta) ELISA Kit | Elabscience, China | E-EL-H0149 |
| PGE2 (Prostaglandin E2) ELISA Kit | Elabscience, China | E-EL-0034 |
| Mouse neutrophil elastase ELISA Kit | Jingmei, China | JM-02886M2 |
| Mouse myeloperoxidase (MPO-DNA) ELISA Kit | Jingmei, China | JM-12706M1 |
| BeyoChIP™ Chromatin Immunoprecipitation (ChIP) Assay Kit with Protein A/G Magnetic Beads | Beyotime, China | P2080S |
| IP-ABE Palmitoylation Kit For WB | AIMS, China | AM10314 |
| Real time-qPCR primers | | |
| Primers for mouse *Il6*  *Forwards:* ОCACTTCACAAGTОGGAGGCTTA  *Reverse:* TGCAAGTGCATCATCGTTGTTC | Accurate Biotechnology, China | N/A |
| Primers for mouse *Il23a*  *Forwards:* CCCCGTATCCAGTGTGAAGATG  *Reverse:* TGAAGATGTCAGAGTCAAGCAGG | Accurate Biotechnology, China | N/A |
| Primers for mouse *Il17a*  *Forwards:* TCAGACTACCTCAACCGTTCCA  *Reverse:* CTTTCCCTCCGCATTGACACA | Accurate Biotechnology, China | N/A |
| Primers for mouse *Rankl*  *Forwards:* AGGGAGCACGAAAAACTGGT  *Reverse:* TACGTCGCATCTTGATCCGG | Accurate Biotechnology, China | N/A |
| Primers for mouse *Atp6*  *Forward:*  TTCCCATCCTCAAAACGCCTAA  *Reverse:*  GAAGTGGGCAAGTGAGCTTTT | Accurate Biotechnology, China | N/A |
| Primers for mouse *Co1*  *Forward:*  TCCCTTGACATCGTGCTTCA  *Reverse:*  AGTCTGAGTAGCGTCGTGGT | Accurate Biotechnology, China | N/A |
| Primers for mouse *Nd1*  *Forward:* GAGCCTCAAACTCCAAATACTCACT  *Reverse:* GAACTGATAAAAGGATAATAGCTATGGTTACTTCA | Accurate Biotechnology, China | N/A |
| Primers for mouse *D-loop*  *Forward:* AATCTACCATCCTCCGTGAAACC  *Reverse:* TCAGTTTAGCTACCCCCAAGTTTAA | Accurate Biotechnology, China | N/A |
| Primers for mouse *LSP*  *Forward:* ATCACCTAAGGCTAATTATTCATGC  *Reverse:*  GAACATGATTTTGTAAAATTTTTAC | Accurate Biotechnology, China | N/A |
| Primers for mouse *HSP1*  *Forward:*  AAATATGACTTATATTTTAG  *Reverse:*  TGCTTTGCTTTGTTATTAAG | Accurate Biotechnology, China | N/A |
| Primers for mouse *HSP2*  *Forward:*  ACGCAATAAACATTAACAA  *Reverse:*  CCCTCTCCTTAAATTTTAAG | Accurate Biotechnology, China | N/A |
| Primer sequences for PCR | | |
| Primers for mouse *Atp6*  *Forward:*  ATACACAACACTAAAGGACGAACC  *Reverse:*  GAGGCTTACTAGAAGTGTGAAAACG | Accurate Biotechnology, China | N/A |
| Primers for mouse *Co1*  *Forward:*  GGAGTCCTAGGCACAGCTCTAA  *Reverse:*  GGAGGGTAGACTGTTCAACCTG | Accurate Biotechnology, China | N/A |
| Primers for mouse *Nd1*  *Forward:*  GCATTCCTAATGCTTACCGAAC  *Reverse:* AAGGGTGGAGAGGTTAAAGGAG | Accurate Biotechnology, China | N/A |
| Primers for mouse *D-loop*  *Forward:* GTGTTATCTGACATACACCATACAG  *Reverse:* TGGGAACTACTAGAATTGATCAGGA | Accurate Biotechnology, China | N/A |
| Primers for mouse *Gadph*  *Forward:*  CCCCTTCATTGACCTCAACTAC  *Reverse:*  GAGTCCTTCCACGATACCAAAG | Accurate Biotechnology, China | N/A |
| Primers for mouse *Actb*  *Forward:*  ATCTGGCACCACACCTTCTACAATGAGCTGCG  *Reverse:*  CGTCATACTCCTGCTTGCTGATCCACATCTGC | Accurate Biotechnology, China | N/A |
| Primers for mouse *Rhoh*  *Forward:*  ATGCTGAGTTCCATCAAGTGCGTGTTG  *Reverse:*  TTAGAAGATCTTGCACTC | Accurate Biotechnology, China | N/A |
| Primers for mouse *Fas*  *Forward:*  TCACCACTATTGCTGGAGTCAT  *Reverse:*  TAAACATCCTTGGAGGCAGAAT | Accurate Biotechnology, China | N/A |
| Primers for *Pg*  *Forward:*  CCCATTCTTTCCCGTTCTCTT  *Reverse:*  CAGCACATCCTTCTTTTCGATG | Accurate Biotechnology, China | N/A |
| Primers for *Pg* *Hum Y*  *Forward:*  GAACGATTTGAACTGGGACA  *Reverse:*  AACGGTAGTAGCCTGATCCA | Accurate Biotechnology, China | N/A |
| Primers for mouse mt-DNA genome  *Forward:* ACTGAAAATGCTTAGATGGATAATTGTA  *Reverse:* GTATGATTAGAGTTTTGGTTCACGGAACA | Accurate Biotechnology, China | N/A |
| Primer sequences for mouse genotyping | | |
| Primers for *Padi4^-/-^*  *P1:*  AGCTTTGTAAGGGGCATCCT  *P2:*  TCAAAGTACCTGATGTGTTGACTG  *P3:*  TGGACCAGTCAGTGAAAGA | Accurate Biotechnology, China | N/A |
| Primers for Ly6G-DTR  *1-F*:  AACTGCTGAGCCATGTCTCC  *1-R*:  TTTTCCCGTGCTCCTCCTTG  *2-F*:  CATCGTGGGGCTTCTCATGT  *2-R*:  ACCCAAACTACCAAGGCCAG  *h-DTR-F*:  TTCTGGCTGCAGTTCTCTCG  *h-DTR-R:*  ACATGAGAAGCCCCACGATG | Accurate Biotechnology, China | N/A |
| Primers for PF4-cre  *oIMR8744:*  CAAATGTTGCTTGTCTGGTG  *oIMR8745:*  GTCAGTCGAGTGCACAGTTT  *oIMR8968:*  CCCATACAGCACACCTTTTG  *oIMR8969:*  TGCACAGTCAGCAGGTT | Accurate Biotechnology, China | N/A |
| Primers for Rosa26iDTR  *oIMR8052:*  GCGAAGAGTTTGTCCTCAACC  *oIMR8545:*  AAAGTCGCTCTGAGTTGTTAT  *oIMR8546:*  GGAGCGGGAGAAATGGATATG | Accurate Biotechnology, China | N/A |
| Primers for *Mettl4^f/f^*  *P3:*  AGCTTTCTAGGTGCTAGGGTT  *P4:*  CCAAAAGACTGTCCTCTGACCT | Accurate Biotechnology, China | N/A |
